# Genome assembly and geospatial phylogenomics of the bed bug *Cimex lectularius*

**DOI:** 10.1038/ncomms10164

**Published:** 2016-02-02

**Authors:** Jeffrey A. Rosenfeld, Darryl Reeves, Mercer R. Brugler, Apurva Narechania, Sabrina Simon, Russell Durrett, Jonathan Foox, Kevin Shianna, Michael C. Schatz, Jorge Gandara, Ebrahim Afshinnekoo, Ernest T. Lam, Alex R. Hastie, Saki Chan, Han Cao, Michael Saghbini, Alex Kentsis, Paul J. Planet, Vladyslav Kholodovych, Michael Tessler, Richard Baker, Rob DeSalle, Louis N. Sorkin, Sergios-Orestis Kolokotronis, Mark E. Siddall, George Amato, Christopher E. Mason

**Affiliations:** 1 Sackler Institute for Comparative Genomics, American Museum of Natural History, New York, New York 10024, USA; 2 Division of Invertebrate Zoology, American Museum of Natural History, New York, New York 10024, USA; 3 Cancer Institute of New Jersey, Rutgers University, New Brunswick, New Jersey 08908, USA; 4 Department of Physiology and Biophysics, Weill Cornell Medical College, New York, New York 10065, USA; 5 The HRH Prince Alwaleed Bin Talal Bin Abdulaziz Alsaud Institute for Computational Biomedicine, New York, New York 10065, USA; 6 Tri-Institutional Training Program in Computational Biology and Medicine, New York, New York 10065, USA; 7 Biological Sciences Department, NYC College of Technology (CUNY), Brooklyn, New York 11201, USA; 8 Biosystematics, Wageningen University, Wageningen 6708 PB, The Netherlands; 9 Illumina Inc. 5200 Illumina Way, San Diego, California 92122, USA; 10 Simons Center for Quantitative Biology, Cold Spring Harbor Laboratory, Cold Spring Harbor, New York 11724, USA; 11 BioNanoGenomics Inc. 9640 Towne Centre Drive Ste. 100, San Diego, California 92121, USA; 12 Molecular Pharmacology and Chemistry Program, Department of Pediatrics, Sloan Kettering Institute, Memorial Sloan Kettering Cancer Center, New York, New York 10065, USA; 13 Department of Pediatrics, Memorial Sloan Kettering Cancer Center, Weill Cornell Medical College, Cornell University, New York, New York 10065, USA; 14 Division of Pediatric Infectious Diseases, College of Physicians and Surgeons, Columbia University, New York, New York 10032, USA; 15 High Performance and Research Computing Group, Rutgers Biomedical and Health Sciences, Newark, New Jersey 07103, USA; 16 Department of Biological Sciences, Fordham University, Bronx, New York 10458, USA; 17 The Feil Family Brain and Mind Research Institute, New York, New York 10065, USA

## Abstract

The common bed bug (*Cimex lectularius*) has been a persistent pest of humans for thousands of years, yet the genetic basis of the bed bug's basic biology and adaptation to dense human environments is largely unknown. Here we report the assembly, annotation and phylogenetic mapping of the 697.9-Mb *Cimex lectularius* genome, with an N50 of 971 kb, using both long and short read technologies. A RNA-seq time course across all five developmental stages and male and female adults generated 36,985 coding and noncoding gene models. The most pronounced change in gene expression during the life cycle occurs after feeding on human blood and included genes from the *Wolbachia* endosymbiont, which shows a simultaneous and coordinated host/commensal response to haematophagous activity. These data provide a rich genetic resource for mapping activity and density of *C. lectularius* across human hosts and cities, which can help track, manage and control bed bug infestations.

## Abstract (web summary)

The common bedbug is a pest for humans, yet its molecular biology is poorly understood. Here, the authors sequence the common bedbug genome and profile gene expression across all life stages to show major changes in gene expression after feeding on human blood.

## Graphical abstract

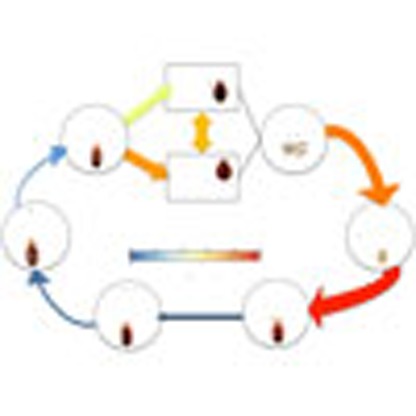

The common bed bug, *Cimex lectularius*, has been associated with humans for thousands of years^1^. There are over 90 described species classified in the family Cimicidae (Order Hemiptera)^2^,^3^, of which three cimicid species are known to be intimate associates of humans: the common bed bug of the temperate regions (*C. lectularius* Linnaeus, 1758), the tropical bed bug (*C. hemipterus* Fabricius, 1803) and the West African species that feeds on humans and bats (*Leptocimex boueti* Brumpt, 1910)^4^. The temperate species analysed in this study, *C. lectularius*, is the most predominant cimicid in densely inhabited human environments such as indoor dwellings and cities.

Several theories exist with respect to how these species made the transition to humans as their primary host^5^. A leading hypothesis posits that the transition occurred when humans lived in caves, where they were exposed to bugs that fed on bats and on other cave-dwelling mammals^1^,^6^. On the establishment of human settlements and dwellings outside caves, human-adapted cimicids accompanied their new host. Given the time that elapsed since this transition, present-day bed bug populations feeding on humans and bats have undergone genetic differentiation in sympatry^7^. Early hunter–gatherer and herder populations migrated over long distances; however, as small towns and villages (and later cities) became established, bed bug infestations grew^8^.

Commerce and human travel then contributed to expanded distributions of bed bugs throughout Europe, Asia and the Americas. European bed bug infestations were recorded in Germany and France by the eleventh and thirteenth centuries, respectively^9^. Bed bugs were also reported in England in 1583 carboxylesterase (CE); however, infestations were uncommon until the seventeenth and eighteenth centuries^1^,^10^. The emergence of heated homes and air travel since the late 1900s has accelerated bed bug infestations in cities globally^11^, as bed bugs could then thrive throughout the year in indoor environments with constant access to blood meals as well as migrate opportunistically and rapidly. Finally, public and commercial locales have been reported to be potentially infested^12^,^13^, as bed bugs are easily transported between their homes and these places by unsuspecting citizens^1^,^5^.

A lull in bed bug infestations began with the introduction of insecticides in the mid-1900s (refs 9, 14); however, a resurgence since the late 1990s and the evolution of insecticide resistance have prompted research to understand the basis of this resistance^15^,^16^,^17^. There is a limited molecular understanding of the biology of the bed bug before, during and after feeding on human blood^18^, which is essential to their life cycle since bed bugs are temporary ectoparasites, whereby they access their hosts for blood feeding and then seek the refuge of the indoor environment for digestion, waste production and mating^9^. To address these questions, we produced the first genome sequence draft of the bed bug, an RNA-sequencing (RNA-seq) functional annotation profile of gene expression across all life stages, and then a full gene annotation and phylogenetic analysis of this important urban pest. In combination with a parallel effort on environmental metagenomics of the highly urbanized and populated landscape of New York City (NYC), we also show that such a draft genome sequence can be used to map and characterize citywide phylogeographic relationships of bed bugs as they interact with their human hosts.

## Results

### Sample collection

**Figure 1:**
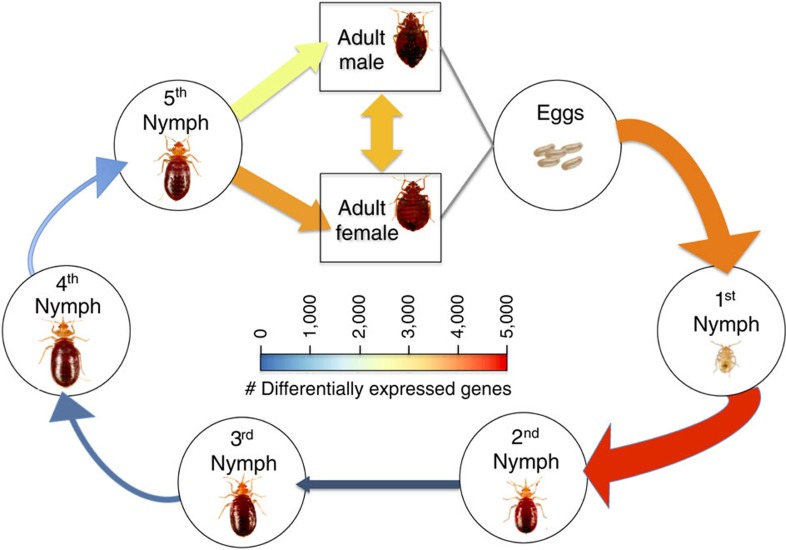
The bed bug life cycle and developmental gene expression profile. The seven stages of the life cycle for *C. lectularius* are shown, starting from an egg and proceeding through five nymphal instar stages, with final differentiation into adult male and female. We used the annotation and RNA-seq data (five individuals per time point in triplicate) to calculate the number of DEGs between all developmental stages and sexes. The DEGs from both adult male and female comprise the arrow from eggs to first instar. The width of the arrows and their colour are proportional to the number of statistically significant DEGs (false discovery rate <0.05, log(fold-change) ≥1.5 and RPKM ≥1).

To build the genome and transcriptome profiles, we extracted DNA and RNA from the standard laboratory Har-73 insecticide-susceptible strain of *C. lectularius*. The bed bug has typical developmental stages of an insect that exhibits hemimetabolous metamorphosis (Fig. 1). Each of the immature stages are nymphal, and there is no pupal stage between the final immature stage and the adult. Blood feeding begins in the first instar nymph stage after hatching from the egg and continues through the next four nymphal instars, followed by the adult male and female. We collected RNA from animals at each of these stages and then sequenced RNA from each collection at each time point in triplicate (Supplementary Table 1). Notably, we collected RNA from unfed bed bugs and recently fed bed bugs to examine the functional genomic profile of the bed bug in relation to blood meals. To avoid human or haemophilic bacterial contamination of the DNA used in genome sequencing and assembly, we used first instar nymphs that recently hatched but had not taken any blood meals.

### Genome assembly

To build the assembly, we used a combination of long and short read technologies. We first created a set of four Illumina TruSeq libraries with insert sizes of 185, 367, 3,000 and 6,000 bp (Supplementary Table 2, see Methods for library details). The combined 252 million paired-end (PE) reads (100 × 100 bp) represented 73 × coverage of the genome and enabled a resolution of small and large genome fragments, with a coverage of 34 × , 12 × , 7 × and 20 × for the four libraries, respectively. A first assembly of the genome was performed using ABySS to calculate accurate insert sizes for our paired reads that could be used for the more-thorough ALLPATHS-LG assembler (Supplementary Fig. 1). The resulting assembly contained 77,082 contigs with an N50 contig length of 12.6 kb. After scaffolding, the assembly consisted of 13,151 sequences with an N50 scaffold length of 945 and 947 kb without and with gaps, respectively. The total estimated length of the first genome assembly was 713.6 million bp (Mb). The full set of assembly statistics from these four insert libraries is listed in Supplementary Table 2.

We then used the Illumina Moleculo kit, which utilizes a unique barcoding and dilution protocol to produce long synthetic reads. After Moleculo software processing, long contiguous reads were created and used in the assembly. The Moleculo sequencing provided 571,913 reads with an average length of ∼3,500 bp (Supplementary Table 3 and Supplementary Fig. 2), with the reads showing a median of >99% (Q30) accuracy. In order to leverage genomic information both from the ALLPATHS-LG and from the Moleculo assemblies, the Metassembler pipeline was used, resulting in a decrease in the number of scaffolds and increase in the N50 length compared to the ALLPATHS-LG assembly, which we then used for the final assembly^19^,^20^. The integrated assembly using the long and short read technologies was a total of 697,867,761 bp from 12,259 scaffolds, with an N50 of 971kb and a N95 of 9.7kb (Supplementary Table 4). We then validated this assembly using the BioNano Genomics Irys system to create 65 × coverage (56 Gb) across single-molecule genome maps from the same strain (Supplementary Fig. 2, see Methods), whereby we observed that 87.44% of the bases in the sequence assembly (*P* value threshold=1e−7) showed accurate orientation and assembly by BioNano molecules. Finally, we used the CEGMA (Core Eukaryotic Genes Mapping Approach) algorithm to establish our coverage of core eukaryotic genes (CEGs, see Methods)^21^. Out of 248 CEGs, the ALLPATHS-LG assembly included 218 completely assembled genes, with an additional 21 CEGs partially assembled, giving us an estimated gene completeness of 96% (239/248).

### Transcriptome assembly and annotation

**Figure 2:**
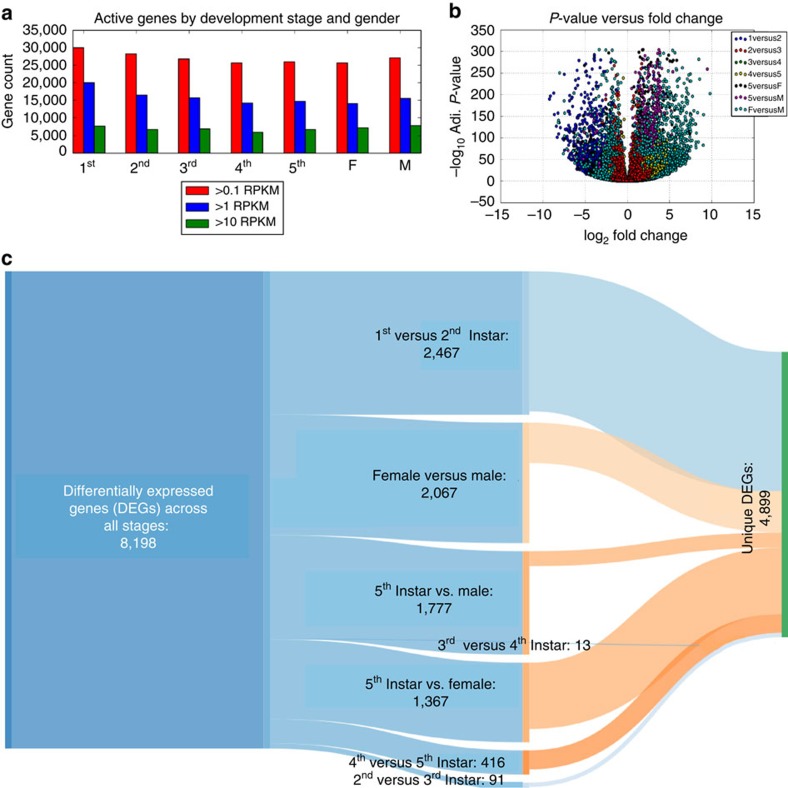
Total and unique genes active in developmental stages. (**a**) Overall transcriptional output and complexity are similar between all the stages of development, yet the number of DEGs is highly variable between different life stages. (**b**) A volcano plot showing the genes with significant differential expression (log fold-change of >2, false discovery rate 0.05 and RPKM of at least 1.0), with the –log_10_ of the *P* value on the *y* axis and the fold-change on the *x* axis. (**c**) A Sankey diagram shows the total number of DEGs for all comparisons (*n*=8,198) between different life stages, and the proportion of each comparison (middle), as well as the DEGs that are unique to each comparison (middle) and those unique across all stages (right, *n*=4,912).

To annotate the genome with the RNA-seq data, we used the MAKER2 package^22^, which utilized both the DNA and RNA sequence data to produce full gene and protein sets. As a reference annotation, we used the pea aphid's (*Acyrthosiphon pisum*), which is a closely related, well-annotated genome sequence^23^. Our genome annotation contains 36,985 unique genes (Supplementary Figs 3–8), which is slightly higher (8–17%) than similar annotated insect genomes such as the pea aphid's genome (Supplementary Fig. 9). While these numbers may indicate a complex transcriptome, it is worth noting that our annotation included RNA-seq data from all life stages of the bedbug and used comparisons to a large set of gene models in *A. pisum*, both of which would create an expansive set of gene models. To gauge which of these genes were developmentally regulated, we first determined the number of genes that were expressed *uniquely* at one stage, and we found that the first instar has a much greater number of unique genes than any other stages (Fig. 1), indicating a burst of specific transcriptional activity on emerging from the embryo. Next, we determined the total number of genes expressed per stage and found fairly consistent gene activity across the stages, with a range of 14,752–20,673 genes detected at levels above one read per kilobase per million reads (RPKM; Fig. 2a,b). However, there is a consistent decrease in overall gene expression and transcriptional activity (number of genes) from the first instar stage through adult.

We then compared gene expression from different parts of the life cycle to detail the stage-regulated changes that occur throughout development (Supplementary Table 5). We used DESeq2 (ref. 24) to discern significant changes in gene expression (fold-change >1.5, and <2, and Benjamini–Hochberg correction for false discovery rate <0.05). We hypothesized that, after a blood meal, a large molecular shift would occur because of ingestion of copious amounts of foreign material. Indeed, we observed a striking increase in the number of significantly differentially expressed genes (DEGs, *n*=4,262 genes) after the first blood meal (between the first and second nymph stages, Supplementary Fig. 2c). Notably, this rapid change in the expression dynamics of the bed bug is the largest change in its entire life cycle, representing 20% of all stage-regulated genes (Supplementary Fig. 2c) and is even larger than the catalogue of sex-regulated genes that distinguish males and females (*n*=2,828 genes).

To address concerns that human DNA or RNA might dominate sequences in the assemblies or DEGs, both transcriptome and genome assemblies were aligned to the human reference genome. Out of 135,489 putative transcripts, only five mapped to the human genome (see Methods). The alignment lengths of these five sequences ranged from 203 to 342 bp, with a per cent identity range of 81–88%. Out of 12,259 genomic scaffold sequences, none mapped to the human genome when requiring an alignment length ≥100 bp and a per cent identity ≥80%. These results indicate that the first instar DNA and RNA collections were indeed before the first human blood meal.

Finally, to assess the repeat content of the genome, we used RepeatMasker^25^ and the Repbase^26^ sets of standard genomic repeats and found that 2.63% of the genome contain repeats. Since RepeatMasker only identifies repeats that have been previously identified in a small number of model systems, we used the RepeatModeler software^27^ to detect and model bed bug-specific repeats and annotated an additional 29% of the genome as repetitive, indicating that many of these newly discovered repetitive elements are relatively understudied and under-represented in current databases. However, similar trends were observed as in other complex metazoans, for example, the most prominent repeats are long-interspersed repetitive elements covering 11% of the genome, with another 2.5% stemming from short-interspersed repetitive elements. A full catalogue of the repeat content of the genome can be found in Supplementary Table 6.

### The functional bed bug microbiome

To investigate the *C. lectularius* microbiome, we performed reciprocal TBLASTX of all *C. lectularius* genes against the full set of bacterial genomes from GenBank (Supplementary Table 7A). The most frequent matching organisms were *Wolbachia*, followed by *Clostridium*. The high prevalence of *Wolbachia* was expected, as they are known to be one of the most prevalent and important endosymbionts of insects^28^,^29^. To create a more conservative set of genes of microbial origin for these sequences, we used a cross-kingdom analytical tool called alien_index^30^ that is designed to determine whether the top BLAST hit for a gene is eukaryotic or microbial^31^. Using the default cutoffs, we found 114 genes that are still strongly predicted to be microbial in origin (Supplementary Table 7B), and the majority were still from *Wolbachia,* indicating a potential function for these bacterial genes.

We next examined the genome of *C. lectularius Wolbachia* endosymbiont (*w*Cle), which was recently sequenced^32^ and found to be essential for *C. lectularius* growth and reproduction by supplying B vitamins^33^. We manually examined the locations of the *w*Cle genes in our assembly and found no evidence of horizontal gene transfer, since bacterial and eukaryotic genes were always grouped on separate contigs. Yet, these *w*Cle genes only appeared as DEGs in two distinct stages: after feeding on blood (1st versus 2nd instar, *n*=70 genes) and immediately after (2nd versus 3rd instar, *n*=11 genes). Consequently, these data suggest a dual host/microbiome response to a blood meal and provide an annotated set of genes linked to the *w*Cle that functions as a putative endosymbiont aide to the blood meal.

When we examined the sequence composition of these genes, we observed that the genes with the highest numbers of single-nucleotide polymorphisms (SNPs) distinct from the *Wolbachia* reference are part of pathways that may have different fitness significances for intracellular symbionts. For instance, the gene *ddl* coding for D-alanine–D-alanine ligase that is involved in peptidoglycan (lipid II) biosynthesis and is required for cell wall synthesis exhibited the highest number of nucleotide differences. The *gltA* gene—a locus frequently used in bacterial and *Wolbachia* phylogenetics and a member of the *Wolbachia* MLST scheme^34^—may be subject to higher rates of recombination^35^,^36^.

**Figure 3:**
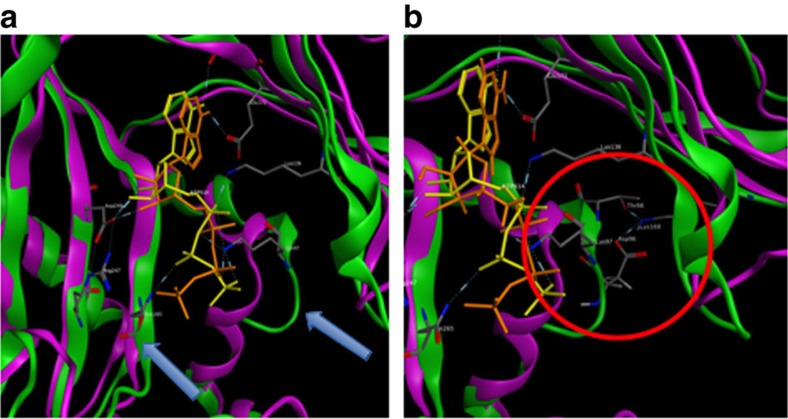
A closeup view of the ATP-binding pocket of DDL. (**a**) Two structure models of the DDL protein, the reference *C. lectularius Wolbachia* DDL (magenta) and the DDL mined from our *C. lectularius* genome sequence (green), are superposed for visual comparison. ATP from a reference model is shown in orange and our protein in yellow sticks. Other amino-acid residues are shown as sticks and colour-coded by chemical element scheme. Arrows indicate a shifted position of the strand 95–98 and a new hydrogen bond between ATP and Asn265 in our protein. (**b**) A new hydrogen bond network in our protein between Thr98, Lys168 and Asp96 is highlighted by red circle.

We utilized protein structural homology modelling to investigate the variations in DDL based on the high-resolution crystal structure of *Escherichia coli* DDLB (PDB ID: 1IOV, 53%), which is the closest related sequence with similarity with the *Wolbachia* DDL^37^. Of the 15 nucleotide differences between the *Wolbachia ddl* gene sequences, two nonsynonymous substitutions are likely to affect protein function (A58D and T84R). Several interesting structural changes in the ATP-binding pocket between wild type and mutant proteins were observed after molecular dynamics (MD) simulations (Fig. 3). Specifically, in the predicted structure of the mutant protein, strand 95–98 was shifted in such a way that potentially allows for more ATP-binding flexibility. As a result, the ATP position was also modified with the formation of an additional hydrogen bond contact with N265, which could lead to an even stronger interaction with the ligand (Supplementary Fig. 10a). Interestingly, this change is facilitated by the replacement of alanine by threonine in position 98 (A98T), thus creating a hydrogen bond network between T98, K168 and D96, which commonly occurred in DDL structures of other species (Supplementary Fig. 10b and Supplementary Table 8).

To further investigate the variability and evolutionary history of *ddl*, we examined multiple *Wolbachia* species (Supplementary Fig. 10). While the two bed bug *w*Cle sequences showed no signs of diversifying selection within the broader taxonomic context, they did harbour eight amino-acid differences. The only codon uncovered to have evolved under diversifying selection was codon 174 (posterior probability=0.92), as evidenced by the consensus of multiple selection detection Supplementary Methods. Over 100 codons in *ddl* were found to be evolving under purifying selection, while the rest were found to evolve under neutrality, thus indicating the evolutionary tendency for this gene to accumulate synonymous substitutions over time.

### Phylogenetic context and activity of anticoagulation genes

Using comparisons with other haematophagous species, we investigated the anticoagulation repertoire of the bed bug. We first aggregated known anticoagulants from 14 other species (see Methods) and used reciprocal BLAST to identify similar genes in the bed bug genome. High-scoring matches for predicted gene products with complete signal peptide secretory sequences were found for three classes of anticoagulant genes and their related proteins, including the serine protease inhibitor infestin, the antihaemostatic (antiplatelet aggregation factor) apyrase and the vasodilator or antihistamine lipocalin; all three are known biological adaptations to blood feeding (Supplementary Table 9). Infestin is a Kazal-type thrombin inhibitor (binding in a slow, tight-binding, competitive process) that is utilized as a structural scaffold template for exogenous anticoagulants^38^. Apyrase promotes the formation of haematomas and is a salivary enzyme that hydrolyses ATP and ADP to AMP and orthophosphate, thus preventing the effect of ADP on haemostasis^38^. Thrombin and intrinsic tenase complex inhibitor ‘lipocalin' has a characteristic eight-stranded antiparallel β-barrel structure that the kissing bug *Triatoma pallidipennis* uses as a scaffold for anticoagulants^39^. Lipocalin is also found in the kissing bug *Rhodnius prolixus.*

Our gene annotation set also found several other characterized proteins with some association to a blood-feeding lifestyle. First, we found orthologues for venom metalloproteases, which are most intensively studied in the context of crotaline and viperine snake envenomations, wherein their haemorrhagic activity relates to endothelial pathology, fibrinogenolysis and their ability to act as disintegrins that inhibit platelet aggregation^39^. In addition, we discovered orthologues for zinc-binding metalloproteases that are also present in the saliomic profiles of a wide range of arthropod sanguivores, including ticks^40^, hookworms^41^ and cimicomorphs related to bed bugs, for example, the reduviids^42^. Serine protease inhibitors are more commonly associated with a blood-feeding habit than are serine proteases^43^. Nonetheless, a variety of these proteases and other trypsin-like plasminogen activators have been characterized from the salivary transcriptomic profiles of the relatively closely related *Triatoma matogrossensis*^42^ and *T. infestans*^44^. Next, we investigated the alignment of our top-matched open reading frames with triatomid bug infestins and homologues in triatomines, which exist in a tandem array of seven paralogues with varying functionalities. A phylogenetic analysis of the infestin proteins (Supplementary Fig. 11) suggests that this *Cimex* protein is indeed a member of the large infesting protein family, which includes dipetalogastin, brasiliensin and infestins. The phylogenetic analysis indicates an evolutionary affinity of the *Cimex* protein with the infestin 1 proteins in other insects.

To create a bed bug-specific set of blood-feeding genes, we filtered predicted gene products against the full non-redundant database of annotated proteins (nr, see Methods). This yielded 161 predicted genes; however, after discarding those that failed to return a weak signal peptide sequence (*D*-score<0.45, see Methods), 28 predicted gene products remained (Supplementary Table 10). Among those predicted to be targeted for extracellular functionality were protein sequences with matches to apyrase, the antithrombin infestin, liocalin, salivary lysozyme and trypsin, a metalloprotease, a carboxylesterase, a high-scoring match to a *Gryllus* gland protein and eight serine proteases. Furthermore, there were four matches to unannotated salivary proteins from *T. infestans*, each with N-terminal signal peptide sequences. Two additional predicted genes of interest to sanguivory are a match to an unannotated 50-kDa midgut protein from blood-feeding sandflies, and an intriguing match to a pig lung surfactant protein. These proteins represent the likely secreted proteins that assist with its haematophagous lifestyle.

### Insecticide resistance

We identified *C. lectularius* orthologues to insect genes known to confer partial or full resistance to insecticides. First, pyrethroids are synthetic organic compounds found in most commercial household insecticides^46^, which can delay the closing of the voltage-gated sodium channel, resulting in prolonged nerve impulse transmission and, eventually, paralysis and death. Nonsynonymous substitutions in the para-type voltage-gated sodium channel gene were first identified in the house fly, *Musca domestica*, termed knockdown resistance (*kdr*), and, specifically in the transmembrane domain II of the sodium channel, have been linked to pyrethroid resistance. This mechanism has also been identified in cockroaches, aphids, mosquitos, cotton bollworm, thrips and various insects extending to different amino-acid substitutions. Bed bugs also harbour *kdr* substitutions that appear to be widely distributed across the United States of America. We identified one voltage-gated sodium channel orthologue in the bed bug genome that exhibited a high degree of sequence identity (86%) and a close phylogenetic match to *kdr* (Supplementary Fig. 12) with a sodium channel protein of the assassin bug (*T. infestans*; Uniprot ID: A0A023F5Z6).

We also found a match to the insect γ-amino butyric acid (GABA)-gated chloride ion channel receptor, which is involved in learning and memory and is encoded by resistance to a dieldrin (*Rdl*) gene that is targeted by insecticides belonging to the cyclodiene (for example, dieldrin) and phenylpyrazole (for example, fipronil) chemical families. A single, nonsynonymous substitution responsible for an Ala-Ser replacement in the second transmembrane domain has been found to confer increased levels of resistance to cyclodienes in *Drosophila* species and other insects, while additional nonsynonymous substitutions have been identified in the same gene in anopheline malaria vectors. *Rdl* gene duplicates have been found in *Drosophila* species and in the green peach aphid to increase gene expression of the resistance-conferring locus, while maintaining the original gene function. Notably, we identified two clusters of GABA receptors in the bed bug genome comprising three and seven proteins each with high similarity (>95% amino-acid sequence match) to other insect homologues.

A third type of putative resistance to insecticides is based on increased metabolic detoxification because of the action of cytochrome P450 monooxygenases (P450), glutathione-*S*-transferases (GSTs) and CEs. GSTs are encoded by a gene superfamily in both arthropod and vertebrate clades, suggesting basic roles in metabolism^45^, and contribute to insecticide resistance by relieving oxidative stress induced by organophosphate compounds. Our reciprocal BLAST analysis (see Methods) detected 16 different bed bug GSTs assigned to various cytosolic classes with a higher representation of sigma class members. Esterases E4 and FE4 capture and hydrolyse organophosphorous insecticides, and their genes can be found in tandem—likely following gene duplication—while being controlled by different regulatory mechanisms^46^. Three homologues (CLG00050, CLG13404 and CLG00055) were found in bed bug with partial identity to other blood-feeding haemipteran insects (kissing bugs, *R. prolixus* and *T. infestans*), nested within the main cimicomorph esterase clade (Supplementary Fig. 13), and we found higher expression of these genes in the last instar and adult stages. These genes represent novel candidates for the molecular basis of insecticide resistance in the bed bug, and it is notable that CE genes are predicted to co-evolve cuticle-thickening genes, which may serve as a first line of defence to insecticides in mosquitos^48^ and bed bugs^49^.

### Phylogenetic contextualization

**Figure 4:**
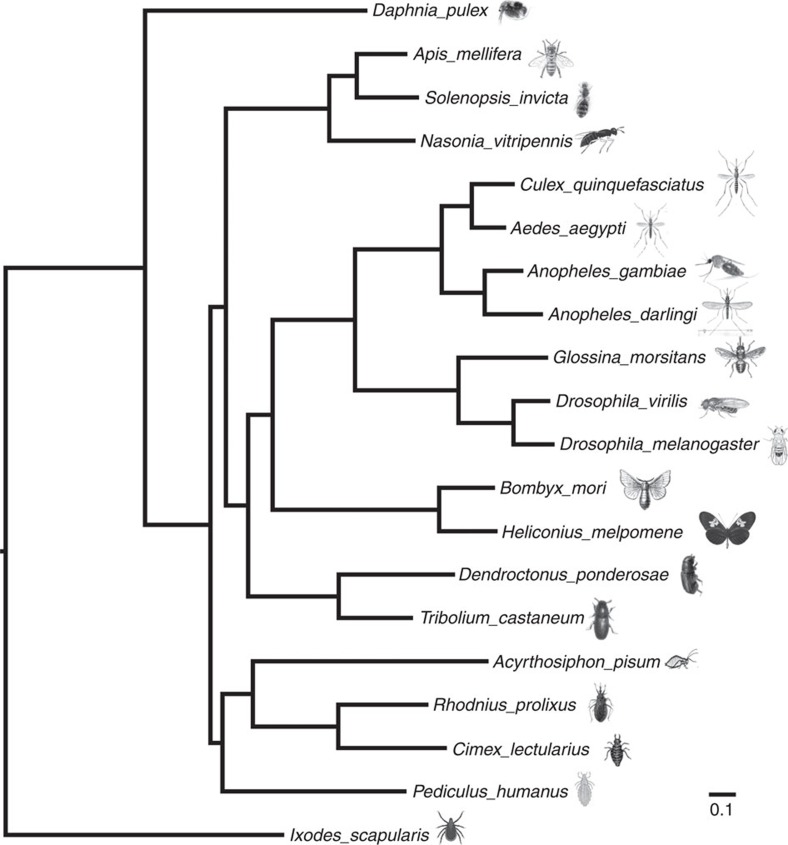
Arthropod phylogenomic relationships. Maximum likelihood estimation of the phylogenetic relationships among genome-enabled arthropods with the blacklegged tick (*Ixodes scapularis*) set as outgroup. All nodes were robust at 100% bootstrap support. The scale bar denotes substitutions per site.

We next investigated *C. lectularius* phylogenetics in two ways. First, we constructed a gene content framework for *C. lectularius* in the context of 20 other fully sequenced arthropod genomes. We analysed this presence/absence of matrix to investigate the phylogenetic relationships of *C. lectularius*^47^,^48^,^49^. Second, we used orthologous gene sequences in phylogenetic analysis to generate an overall hypothesis of relationships based on sequences. The gene content matrices were analysed using equally weighted parsimony, Dollo parsimony and maximum likelihood using the BINGAMMA model in RAxML^50^. The proteome sequence matrix was analysed using maximum likelihood in RAxML implementing the general time-reversible substitution model estimated from our arthropod sequence data (see Methods). A total of 11,919 orthologous protein sequences were established among the 20 fully sequenced arthropod genomes. The analyses of both the gene content data (Supplementary Fig. 14) and the sequence data (Fig. 4) yielded broadly congruent results with the protein sequence analysis being entirely in agreement with the accepted topology of insect relationships while also being fully robust (bootstrap support 100% at each node). These phylogenetic relationships place the bed bug closest to the *R. prolixus*, and then to *P. humanus*, both of which are blood-feeding haemipterans known to associate with humans.

### Urban phylogeography of bed bugs

To investigate the diversity of bed bugs across NYC, we utilized the PathoMap resource^51^ that performed metagenomic sampling of 1,447 locations across NYC, including 465 subway stations of the NYC Metropolitan Transit Authority. This project swabbed each location and then performed shotgun sequencing on the resulting DNA. The project was primarily intended to look at microbial diversity across NYC. However, because of the nature of the sample collection, DNA from any taxa present in the subway, or carried on individual's shoes into the subway, would be found. We aligned all of the reads for all locations to our reference bed bug genome using Burrows-Wheeler Aligner^52^ and called variants using freebayes^53^. We then filtered the data to only include variants having calls for 90% of the sites. The bed bug SNP matrix was used to construct phylogenetic trees for the different subway lines and for divisions based on location (aboveground and belowground) and borough of NYC where the sample was obtained.

**Figure 5:**
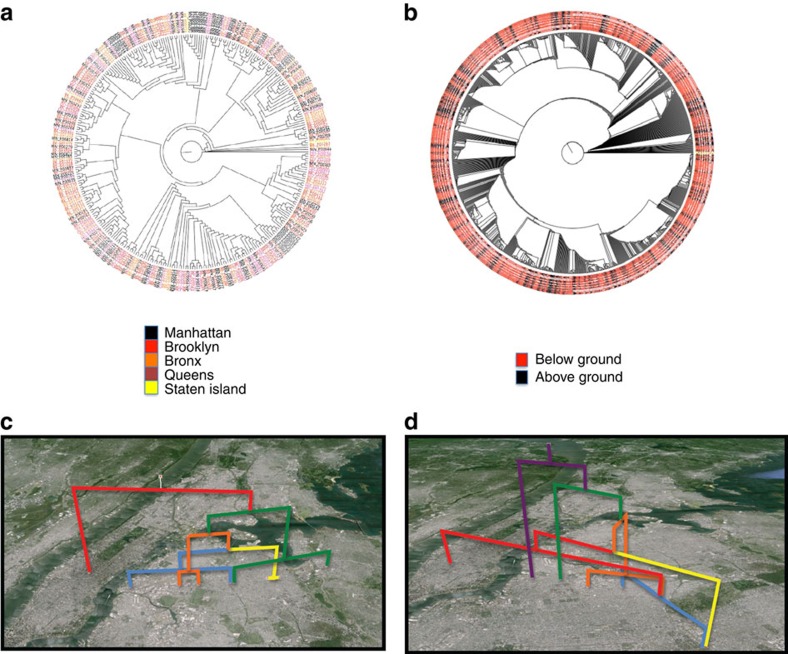
Phylogeographic distribution of bed bug DNA across New York City. (**a**) A comparison of the DNA found on subway benches across different boroughs. (**b**) A comparison of the DNA found aboveground and belowground. (**c**) The seven subway line and (**d**) the L subway line bed bug relationships are overlaid on a map of New York. The phylogenetic subgroup (colours) showed the same branch point for these two lines, both exhibiting an early split between the red and yellow subgroups across the different areas of the city.

We first sought to determine whether there was any biologically meaningful phylogenetic structure in the SNP data sets. Accordingly, we examined the trees for structure relating to several variables: above/belowground, borough, object swabbed (for example, benches and turnstiles) and material swabbed (for example, metal and plastic). Following visualization, the retention index (RI) of each variable was calculated for each tree. A randomization test was then conducted for each variable, testing whether or not the actual RI was greater than the RIs of randomized data. The only variables showing significant structure are borough (Fig. 5a) and above/belowground (Fig. 5b). Although significantly different from randomized null distributions, the borough (RI=0.1969; *P*=0.0212) and above/belowground (RI=0.1870; *P*=0.0024) characters are highly variable on the tree, showing numerous small patches of structuring rather than a few gains and losses of each character. These results likely relate to the potential largely panmictic (random mating) nature of these populations and limitations of the sequence data, including the potential cross-mapping to a related species. We next visually examined whether the subway lines themselves exhibited phylogenetic structure. The two East-West lines of the NYC subway system (Fig. 5c–d) showed a similar phylogeographic structure, with the same split re-occurring in both lines and subsets of the variants staying within one borough. This structure suggests that areas of the city in close proximity to each other show bed bug populations that are related to each other, and one borough's population can be distinct from others.

## Discussion

These data represent the first genome and transcriptome assembly, mapping and functional characterization of the *C. lectularius* (bed bug) genome. The gene expression profile of the bed bug demonstrated that the first blood meal of the bed bug is the most dynamic period of the bed bug's transcriptional activity, and thus has broad implications for Supplementary Methods that may target these haematophagic pathways and mechanisms. Indeed, the discovery of a secreted prolylcarboxypeptidase is intriguing in light of the association with angiogenesis^54^ and the ability to activate prekallikrein^55^. In contrast, other insect prolylcarboxypeptidases are more active in the midgut of insects than in salivary secretions^56^. Predicted protein sequences similar to the accessory gland protein of *Gryllus veletis*, a cuticular protein potentially associated with salivary ducts, are believed to be involved in the prevention of microbial putrefaction of cimicomorph blood meals^42^,^52^. Similar functions are anticipated for high-copy transcripts in other sanguivorous invertebrates^57^.

Although we observed 8,198 genes with stage-specific regulation, there was also some overlap between different stage-by-stage comparisons (Fig. 3), such that we annotated a total of 4,912 unique, significant DEGs across all life stages. This means that, although the majority of the 38,615 annotated genes were active at some point during development, only 13% of genes demonstrated significant differential expression between stages. This matches results from other studies, which have shown that much of the differential gene regulation is cell- or tissue-specific, or exhibits a finer-resolution^60^; for example, *Drosophila melanogaster* expression can change at an hourly rate during development and changes dramatically by cell type^61^,^62^. Thus, while our genome annotation is estimated to be very complete at the gene-count level, as estimated by CEGMA's CEG count (96%), and in comparison with other species, undoubtedly additional tissues, cell types and substages can now be characterized to classify other genes' spatiotemporal activity and putative function.

The *C. lectularius* genome's orthologous gene content, repetitive element structure and overall gene number further contextualize the organism within insect phylogenetic history, and include a comparable or larger annotation set than most insect species (Supplementary Fig. 8). With an analysis of 20 fully sequenced arthropod genomes, leveraging both gene content and sequence data, we estimate that our phylogenetic assignments are congruent and accurate (Fig. 5 and Supplementary Fig. 10). In this context, all of the major orders of insects that could be included in the analysis, that is, Diptera, Coleoptera, Lepidoptera, Hemiptera and Hymenoptera, are robustly monophyletic. The internal relationships within orders with more than two taxa (Hymenoptera, Diptera and Hemiptera) matched existing phylogenetic assessments, and the placement of Hemiptera as the sister group to the rest of insects was also in accord with the accepted understanding of insect order systematics^63^. These closely related species provide further clues about the evolutionary history and phylogenetic relationships of the bed bug, which can help direct future studies and genome assembly prioritization.

Interestingly, we were able to show that metagenomic data created for one purpose can have broad uses and can be repurposed on the completion of a new genome. The bed bug genetic diversity we found along the NYC subway metagenome indicates that geography can shape the distribution of genetic variants in the landscape and may serve as a ‘molecular echo' of the species in the city, and their DNAs' movement by human hosts^53^. Multiple ongoing efforts for environmental sampling and shotgun-based DNA-sequencing exist, such as those from the MetaSUB project (http://www.metasub.org), which are advancing our understanding of species diversity and complexity in rural, remote and urban settings. Yet, an ongoing challenge for data from such metagenomics profiling is the ‘missing genomes' problem, such that we know that databases are incomplete and that it may not be possible to always match genomic DNA data to the correct genome. As more reference genomes become available, such as this one for *C. lectularius*, we have shown here that orphaned reads from metagenomics projects can be successfully ‘rescued' and mapped to a reference genome that previously were unknown. Furthermore, such alignments can be used to discern a potential phylogenetic relationship between strains in a city, which can then aid in an understanding of the differences between strains for pest control and characterization.

Historically, various methods of resistance to insecticides have been documented, including sodium channel blockers, enzyme detoxification pathways and thicker chitin layers that prevent insecticide penetration of the outer exocuticle. Notably, we see evidence of many of these same mechanisms present and active in the bed bug, with increased activation after feeding on blood meal. The information presented here provides an essential biomolecular resource that can aid in understanding the origin, development and genetic basis of resistance to insecticides, as well as in providing a baseline for population-level comparisons. In turn, this knowledge will help control bed bug infestations, improve management of species diversity and lead to a greater understanding of the fundamental biology of this ancient, eukaryotic ‘companion' of humans.

## Methods

### Raw sequence data

The genome assembly validated by the NCBI, where it was checked for adaptors, primers, gaps and low-complexity regions. The genome assembly has been approved and given the accession number JRLE00000000 and BioProject PRJNA259363. All genome-sequencing data have been deposited in the SRA with accession number SRS749263. RNA-seq data are available as FASTQ files and were quality-checked and deposited in the SRA with accession number SRR1790655.

### Bed bug samples

The bed bugs were taken from a Harlan strain colony maintained by Louis Sorkin (American Museum of Natural History). The Har-73 strain was originally collected by Harold Harlan in 1973 from an infestation at the US Army barracks in Fort Dix, NJ, USA, and has been raised as a laboratory pesticide-susceptible strain since that time. Bed bugs were reared in ∼236.6 ml (8 oz) glass canning jars, where the metal covers had a 250–350-μm hole mesh-screening heat-glued on the inside. Heat glue was applied to the outer circumference of the screen surface to leave a 3-cm-diameter central circle of exposed screen. Folded cardboard was used as substrate. Jars were inverted on a human arm for feeding for 30 min on a monthly basis. Jars were kept in plastic box with an open lid and left at room temperature. Specimens used for nucleic acids extraction were 1st instar nymphs that recently hatched but had not taken any blood meals (∼1 mm in length, pale to white in colour).

### Transcriptome assembly

The bed bug transcriptome was produced using the Trinity assembler r2012–10–05 (ref. 59). To reduce the amount of redundant information fed to Trinity, duplicate sequences among the 631,227,170 50-bp single-end reads were removed using the fastq-mcf programme from the ea-utils library. This was achieved using the command line options -0 -D 50 n/a. Before assembly, the adapter sequencers were trimmed from all reads using SeqPrep v1.0 (ref. 21) with the following parameters: 5′-AAGATCGGAAGAGCACACGTCTGAACTCCAGTCACBAGATCGGAAGAGCGTCGTGTAGGGAAAGAGTGTA-3′. Basecall quality trimming was then performed using SolexaQA^60^ with a phred score cutoff of 20 (-h 20) in DynamicTrim.pl and a minimum trimmed read length of 23 (-l 23) in LengthSort.pl. Trinity was run with the following parameters: --seqType fq --JM 200G --CPU 32. The assembly statistics are shown in Supplementary Table 9.

### CEGMA and sequence data validation

CEGMA v2.4.010312 (ref. 20) was used to check for the existence of CEGs in both the genome and transcriptome assemblies. Default parameters were used for the genome assembly, while --max_intron 0 was used for the transcriptome assembly. To assess the validity of the final assembly, the CEGMA^20^ was used to establish our coverage of CEGs. Out of 248 CEGs, the ALLPATHS assembly included 218 completely assembled genes, with an additional 21 CEGs partially assembled, giving us an estimated gene completeness of 96% (239/248). We also had the genome assembly validated by the NCBI, where it was checked for adaptors, primers and low-complexity regions. The genome assembly has been approved and given the accession number JRLE00000000 and Bioproject PRJNA259363, and all the RNA-seq data have been deposited in the SRA (ID:264998).

### Functional annotation

We performed functional annotation of bed bug sequences based on the gene ontology (GO) vocabulary using the Blast2GO v2.5.0 pipeline (https://www.blast2go.com) with the following parameters: java -Xmx50G -cp *:ext/*: es.blast2go.prog.B2GAnnotPipe -in bedbug.allBBgeneMatches.txt -out bedbug_out_50G.annot -prop b2gPipe.properties.local –annot, where b2gPipe.properties.local points to a local Blast2GO database. We also used InterProScan v5.5–48.0 (ref. 61) with the following parameters: -dp -f TSV,XML,GFF3 -goterms -iprlookup -i Cimex_lectularius.

### Human contamination of RNA-seq data

Unaligned reads retained when producing previously described RNA-seq alignments to the Metassembler genome assembly were aligned to human genome hg19 using STAR. The samtools view command was used to count aligned reads with the -S -c -F 4 options.

### Active gene discovery

Sorted bam files for each developmental stage and sex, as described previously, were used as input to the rpkmforgenes.py programme^62^. Each replicate bam file was processed separately. The resulting RPKM values were filtered at three different RPKM thresholds: 0.1, 1 and 10. A gene model is only considered active in the case that RPKM values for all three replicates surpassed the threshold. The counts for genes considered active were plotted using Python's matplotlib.

### Analysis of genes related to blood-feeding activity

Several suites amino-acid sequences from anticoagulants and other bioactive proteins involved blood feeding known from other sanquivorous taxa were prepared as target databases for blastp searches using unannotated predicted gene products from the combined Qmolecula/allpaths hybrid assembly. Those targeted were antithrombins, factor Xa inhibitors, platelet aggregation and activation inhibitors, hyaluronidases and plasminogen activators. In addition, the full set of predicted gene products was compared both with ToxProt, a compilation of all toxin proteins produced by venomous animals, as well as a third query database comprising all salivary protein sequences already annotated for Cimicomorpha at NCBI. The latter consists primarily of those sequences available for the saliome of Tratima infestans. High-scoring matches (*e*-value<−60) then were sorted and evaluated for relevance to salivary and blood-feeding-related functionality. Premised on the notion that to be biologically active in the context of sanguivory, and that they would be expected to be targeted to the extracellular environment, amino-acid sequences were subjected to the prediction of N-terminal signal peptide regions (*D*-cutoff=0.50), leveraging artificial neural network systems through SignalP 4.1 at http://www.cbs.dtu.dk/services/SignalP. Predicted gene products were then compiled and compared with BLASTP against the full suite of available annotated sequences (NR in GenBank) to determine whether another non-target functionality was a better match; if a better *E*-value was found these were removed.

We mined the set of bed bug protein sequences via BLASTP by using as queries a multitude of proteins from other species known to confer partial or full resistance to insecticidal compounds, when (1) containing one or more amino-acid replacements, (2) their genes are duplicated or (3) their genes are associated with transposable elements. The bed bug hits were queried themselves against the UniProt protein knowledgebase (http://www.uniprot.org) using BLASTP, and the results were manually inspected for similarity to candidates of known function.

### Bacterial genetic traces

We downloaded all of the complete bacterial genomes that were listed in Ensembl release 24 (ftp://ftp.ensemblgenomes.org/pub/release-24/bacteria/fasta). In total, this sample included 20,030 bacterial strains. We ran reciprocal TBLASTX searches between the bacterial genomes and both the *C. lectularius* gene set and the full genome sequence using a cutoff *E*-value of <1e−5 and required a 30-bp overlap match. For the SNP calling, we ran MUMmer^63^ to compare the gene calls from the bed bug genome against the reference *C. lectularius Wolbachia* endosymbiont (*w*Cle) genome^32^.

### Protein modelling

Protein structural modelling was carried out with SWISS-MODEL (http://swissmodel.expasy.org) producing a high-quality structure with a model-template C-α root mean square deviation of 2.3 Å. The models were further refined with MD simulations with the Amber14 MD suite^64^. The proteins and ATP molecules were placed in a water box, and after initial minimization and equilibration for 1 ns, the production run with the canonical (NVT) ensemble and Langevin thermostat heat exchange totalling 100 ns was conducted on a high-performance Linux cluster with NVIDIA Tesla GPU nodes. MD trajectory files were collected and an average structure over all 100-ns timeframes was calculated for each model with the VMD programme^65^ and followed by a brief minimization. Post-MD simulation analysis and visual representations were conducted in MOE programme^66^.

All available 39 X-ray crystal structures of DDL proteins were downloaded from the Protein Data Bank (http://www.rcsb.org). After aligning protein sequences, we searched for the residues that were located in the same positions as in the reported network, and indeed found substantial supporting evidence for such network occurrence. Among these 39 structures, 24 of them have Lysine in the position similar to K168 of *Wolbachia*. Aspartic acid in position 96 is conserved among 38 available crystal structures. There are some variations in position 98, where we also observed a mutation A98T. Aspartic acid is the most common amino acid in this position (occurred 15 times), followed by Leucine (also 15 times). There is no available crystal structure of DDL with threonine in position 98 (Supplementary Table 8). Interestingly, three-member networks similar to the D96-T98-K168 hydrogen-bonding network observed after MD simulations in the K168 mutant form of *Wolbachia* were present in all D96-D98-K168 and D96-L98-K168 X-ray crystal structures. However, if K168 is replaced with E, as happens in 10 crystal structures, then such a network is not observed. It is especially evident for sequences where position 98 is occupied by amino acids with aliphatic side chains, for example, Leucine. We found it very intriguing that such a hydrogen bond network occurred only in the mutant protein despite the fact that our template structures, 1IOV and 4C5B, lack this network. As mentioned in the main text, the replacement of Alanine with the larger Threonine side chain, which can serve as a hydrogen bond donor, may help in the formation of this three-member network T98-D96-K168 and facilitate the shift of T98 towards K168 in the mutant protein that resulted in 95–98 strand shift and create more space for ATP binding in the mutant DDL versus wild-type A98 DDL.

On the basis of the computational model we concluded that among eight observed mutations, A58D, I60V, T84R, I93V, A98T, L104F, G108D and I109V, none was directly involved into the binding of ATP. However, it is worth noting that, in the wild-type protein, the residues in positions 58, 60 and 84 are in close proximity and form a hydrogen-bonding network that stabilizes loop formation in this region. It was expected that a change from a small neutral residue to a larger charged residue (for example, A58D and T84R) might cause reorganization of the loops. The comparison of the wild-type and mutant DDL models suggests that a replacement to oppositely charged amino acids might lead to stronger interactions within this network. In addition to hydrogen bonds, strong ionic interactions occur between D58 and R84 in the mutant protein. This in turn leads to partial changes in adjacent flexible regions as seen in Supplementary Fig. 10 and may cause some alterations in ligase activity.

## Additional information

**Accession codes**: The genome assembly has been deposited in NCBI under the accession code JRLE00000000 and BioProject PRJNA259363. All genome-sequencing data have been deposited in the SRA with accession code SRS749263. RNA-seq data have been deposited as FASTQ files in the SRA under accession code SRR1790655.

**How to cite this article**: Rosenfeld, J. A. *et al.* Genome assembly and geospatial phylogenomics of the bed bug *Cimex lectularius*. *Nat. Commun.* 7:10164 doi: 10.1038/ncomms10164 (2016).

## Disclaimer

The content is solely the responsibility of the authors and does not necessarily represent the official views of the NIGMS or the National Institutes of Health.

## Supplementary Material

Supplementary Information Supplementary Figures 1-14, Supplementary Tables 1-10, Supplementary Methods and Supplementary References
