# Supplementary material for: Genome assembly and geospatial phylogenomics of the bed bug Cimex lectularius
Source: Nat Commun. 2016 Feb 2;7:10164. doi: 10.1038/ncomms10164 (PMC4740774; doi:10.1038/ncomms10164)
Supplement: Supplementary Information — Supplementary Figures 1-14, Supplementary Tables 1-10, Supplementary Methods and Supplementary References [file ncomms10164-s1.pdf]

Supplementary Figures

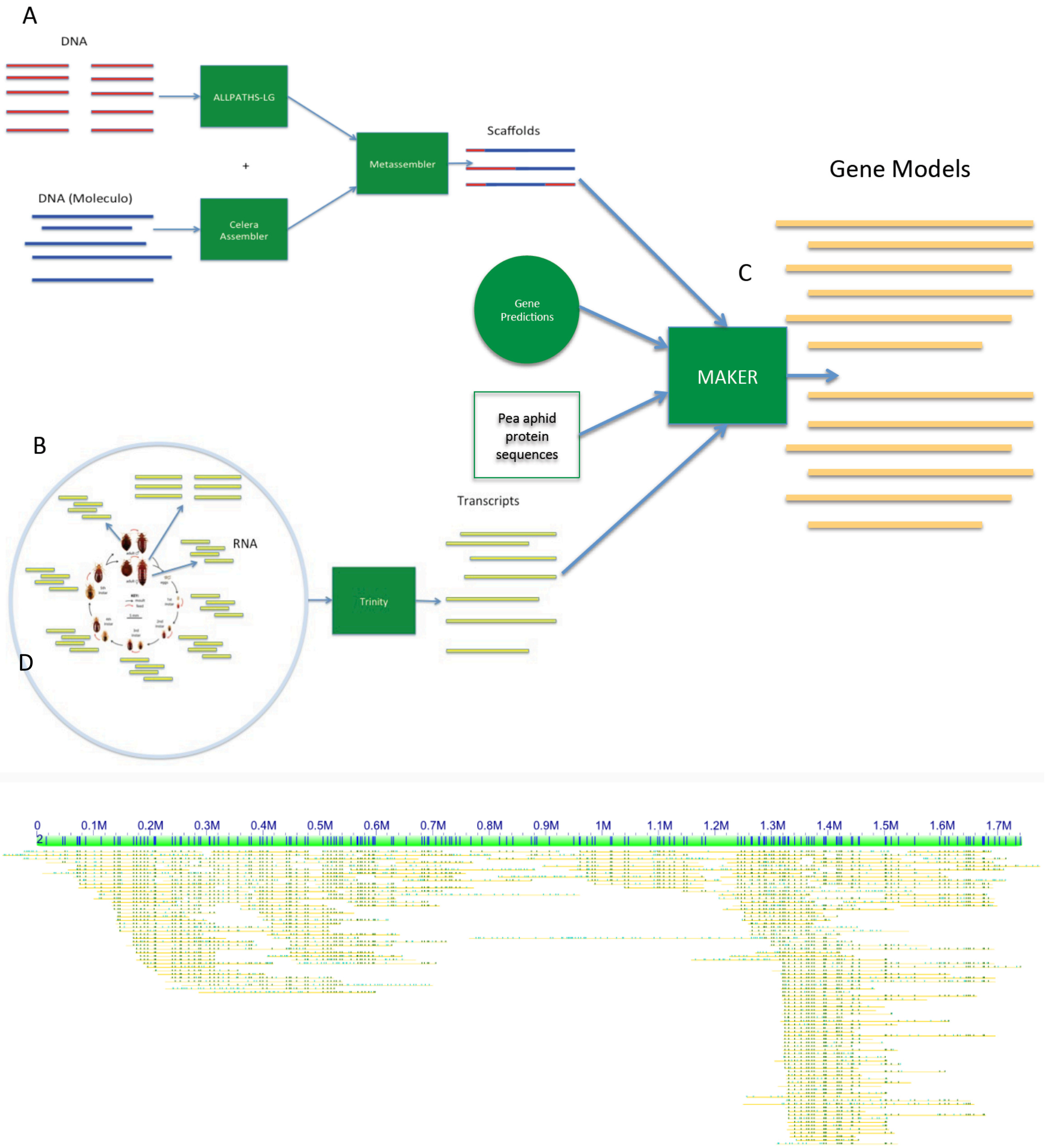

Supplementary Figure 1. Genome sequence assembly and annotation pipeline. (A) Both short (Illumina paired-end libraries) and long read (Moleculo) methods were used to create raw data from DNA extracted from first instar nymphs prior to blood feeding. Reads were analyzed with ALLPATHS-LG and the Celera Assembler, respectively, and then merged with Metassembler to produce final scaffolds. (B) RNA-seq data from all developmental stages created with 50bp single-end reads, as well as one developmental stage with 100bp paired-end reads for improved assembly with Trinity. (C) Trinity transcripts combined with the pea aphid proteome and gene predictions fed into MAKER to create the final gene models and GTF files. (D) Single-molecule genome maps (yellow bar highlighting the DNA backbone, dark green labels showing aligned labels, and cyan labels showing unaligned labels) were aligned against the in-silico motif map of scaffold CLS00019.1 (green bar highlighting the scaffold, black bars showing the predicted label positions). Strong support across the entire 2.8-Mb sequence scaffold.

A

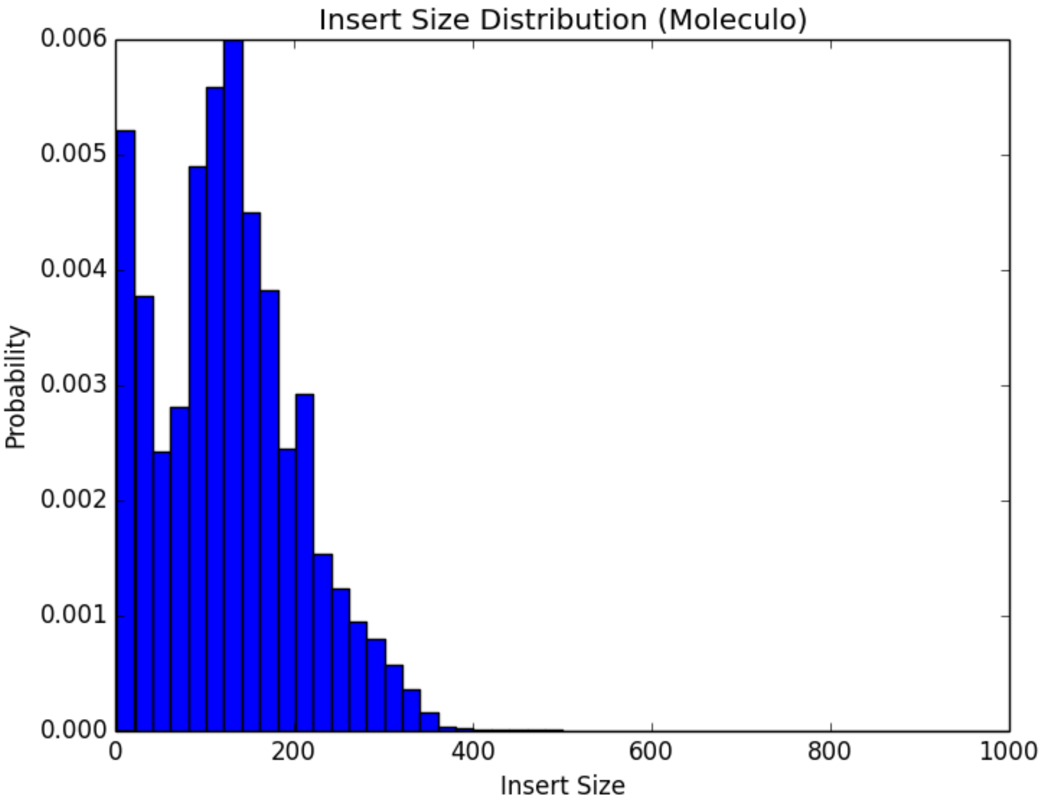

B

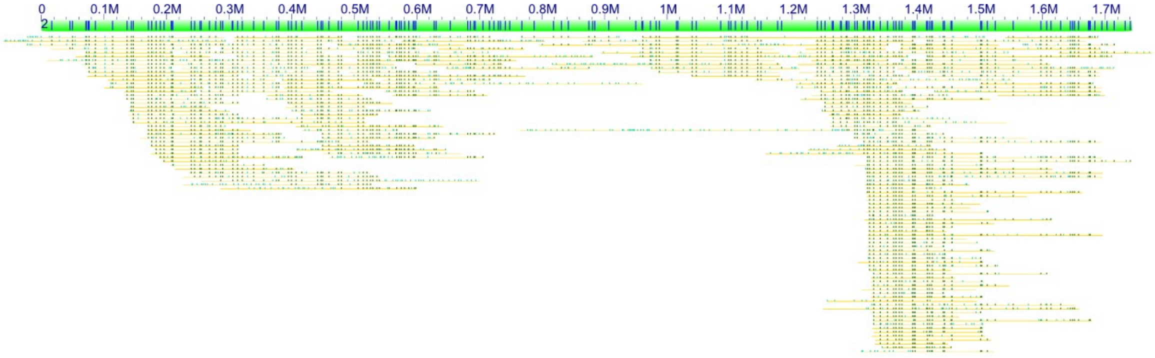

Supplementary Figure 2. Genome assembly validations (A) Distribution of overlapping paired read insert sizes based on Moleculo alignment. We plotted the proportion (*y*-axis) of fragments with varying estimated insert sizes (*x*-axis) from the alignment of short reads to the Moleculo long reads. These results show that the 185bp library was close to the expected size range for the assembly. (B) Single-molecule genome maps were aligned against the in-silico motif map of scaffold CLS00080. There is broad single-molecule support across the scaffold, but weaker support at around 0.8-0.9 Mb and around 1.2 Mb.

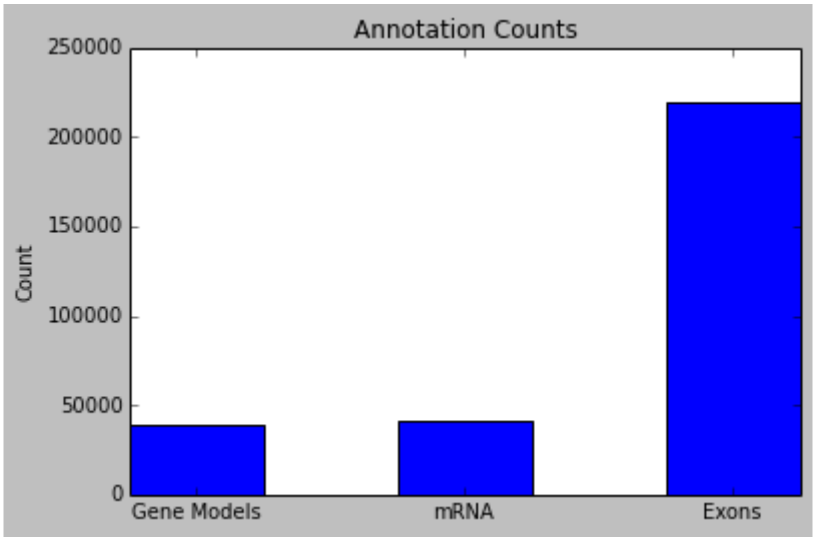

Supplementary Figure 3. Summary of gene features from the assembly. Data show the total number (*y*- axis) of various features from the MAKER-based genome sequence annotation, including gene models, mRNAs, and total number of exons (*x*-axis).

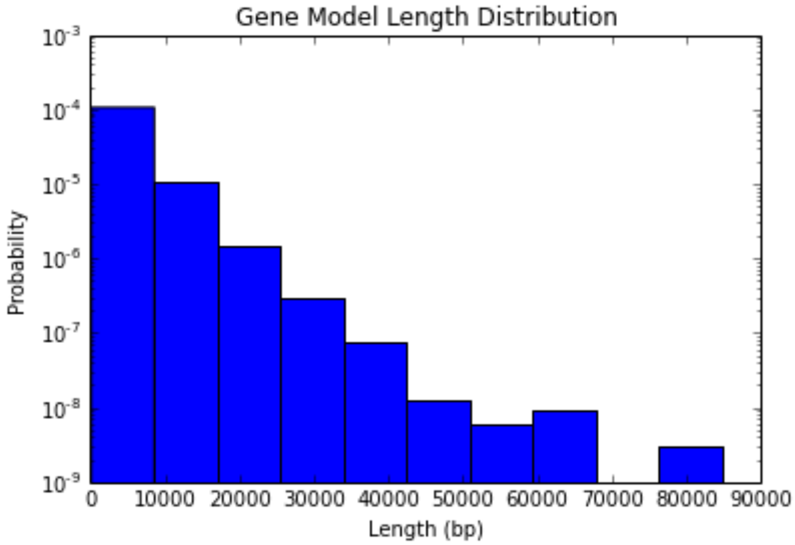

Supplementary Figure 4. Distribution of gene model sizes. The distribution of lengths for genes at varying bins (*x*-axis) is plotted as a function of their count (*y*-axis).

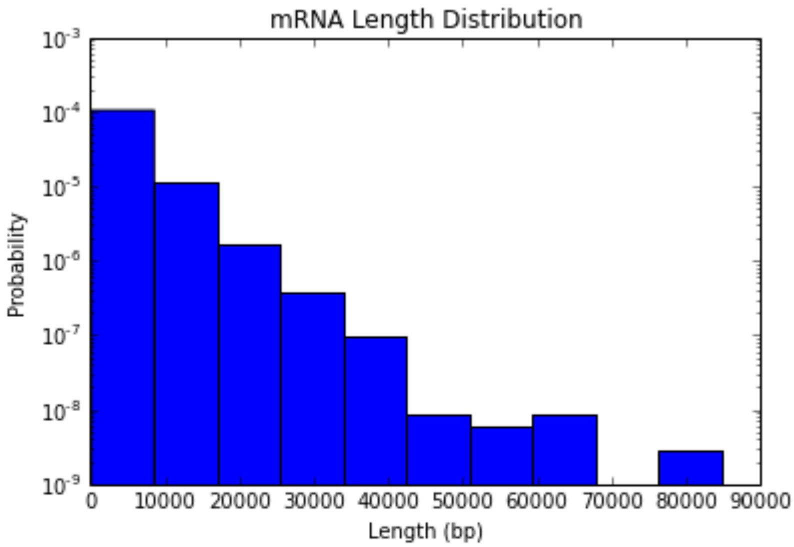

Supplementary Figure 5. Distribution of mRNA model sizes. The distribution of lengths for mRNAs at varying bins (*x*-axis) is plotted as a function of their count (*y*-axis).

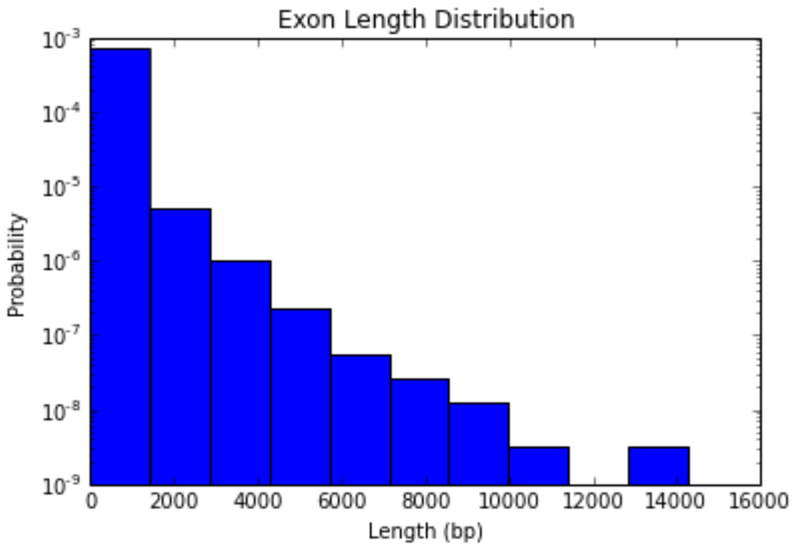

Supplementary Figure 6. Distribution of exon sizes. The distribution of lengths for exons at varying bins (*x*-axis) is plotted as a function of their count (*y*-axis).

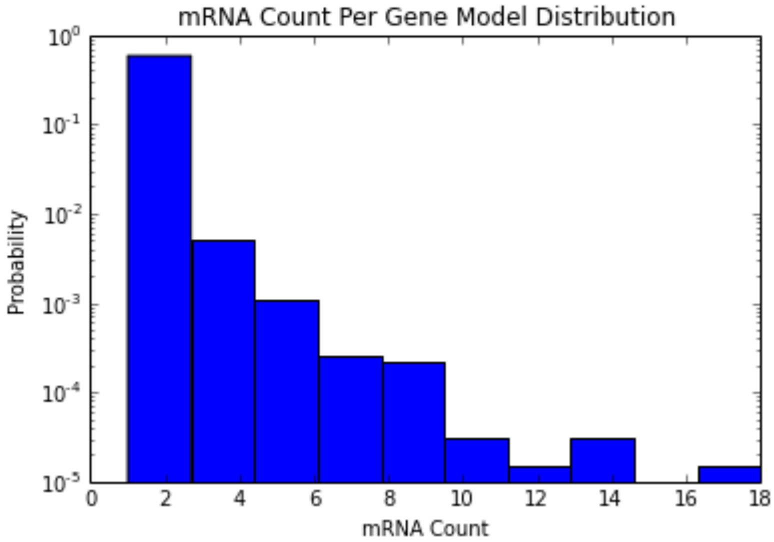

Supplementary Figure 7. Distribution of mRNA counts per gene. The distribution of counts of predicted mRNAs for each gene (*y*-axis) is plotted as a function of their bin (*x*-axis).

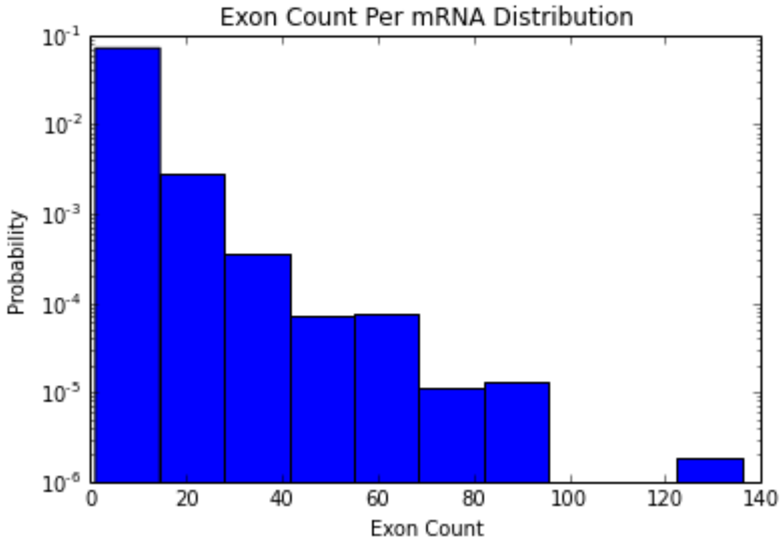

Supplementary Figure 8. Distribution of exon counts per gene. The distribution of counts of predicted exons for each gene (*y*-axis) is plotted as a function of their bin (*x*-axis).

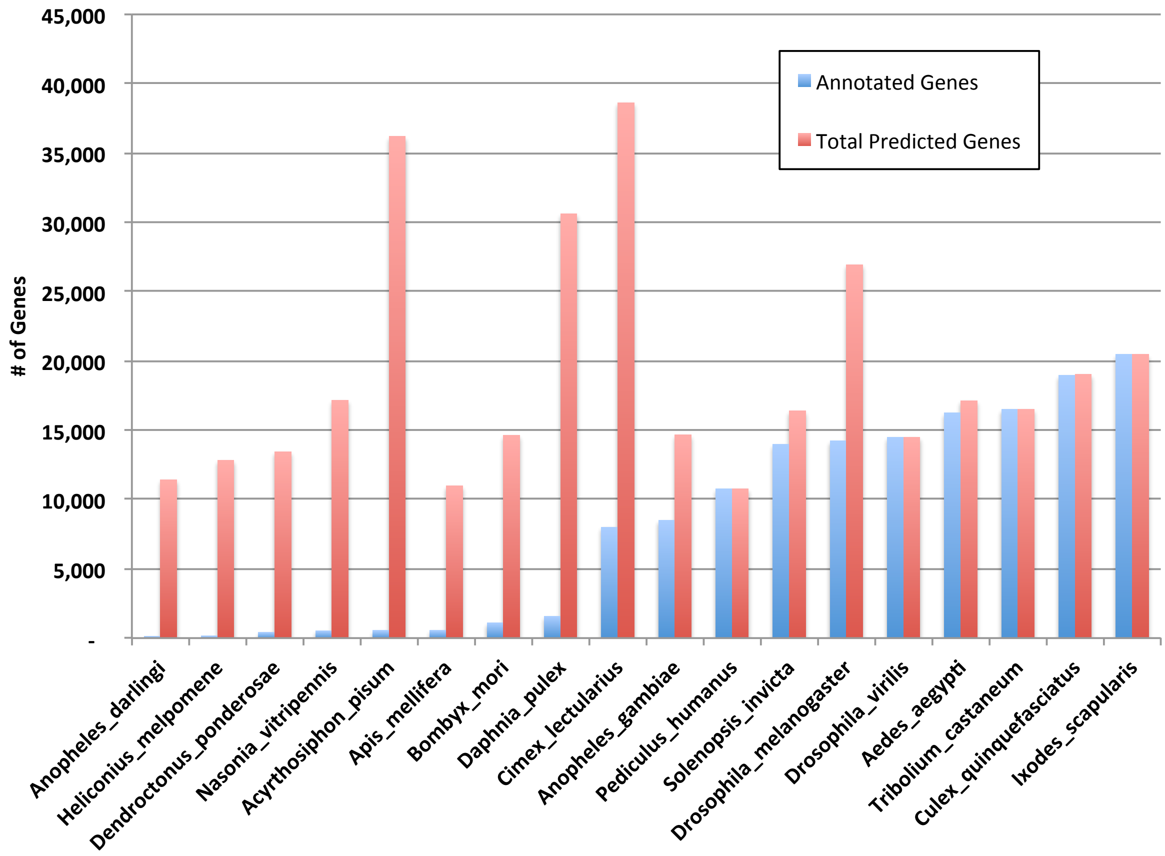

Supplementary Figure 9. Comparison of *Cimex lectularius* genome annotation relative to other arthropods. Number of annotated genes (blue) and the full set of genes (red) of the *C. lectularius* genome, showing the highest number of genes and an average number of annotated genes by UNIPROT.

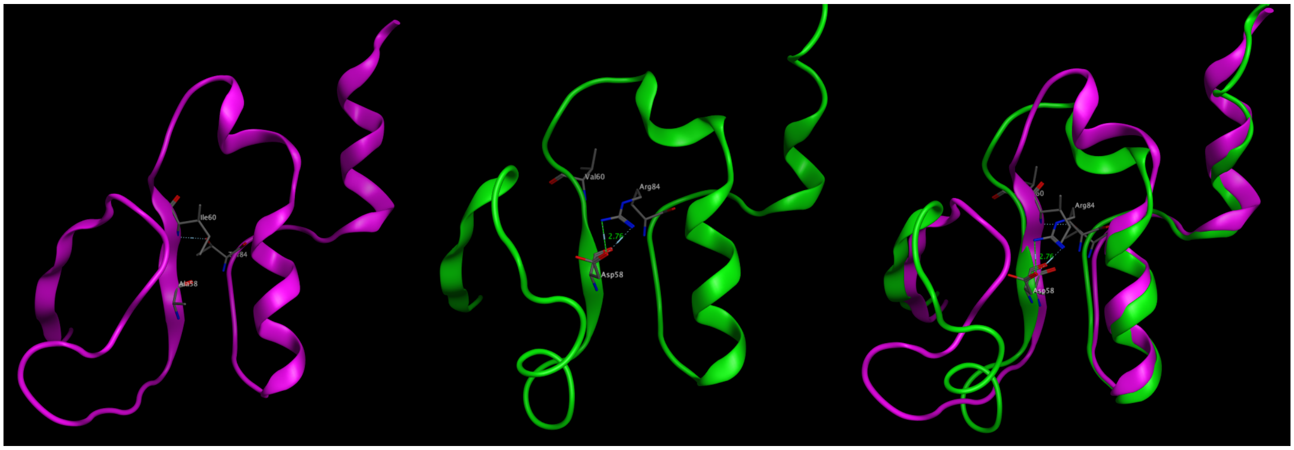

A B C

Supplementary Figure 10. DDL protein three-dimensional structural modeling. (A) wild type (magenta), (B) mutant (green), (C) structural model superposition. Based on computational modeling none of the eight observed amino acid substitutions (A58D, I60V, T84R, I93V, A98T, L104F, G108D, I109V) was directly involved in ATP binding. In the wild type protein, residues 58, 60 and 84 are in close proximity and form a hydrogen-bonding network that stabilizes loop formation in this region. The expectation was that a change from a small neutral to a larger charged residue (e.g. A58D, T84R) might cause reorganization of the loops. The comparison of the wild type and mutant DDL structural models suggests that a replacement to oppositely charged amino acids may lead to stronger interactions within this network. In addition to hydrogen bonds, strong ionic interactions occur between D58 and R84 in the mutant protein. This, in turn, leads to partial changes in adjacent flexible regions and may cause some alteration in ligase activity.

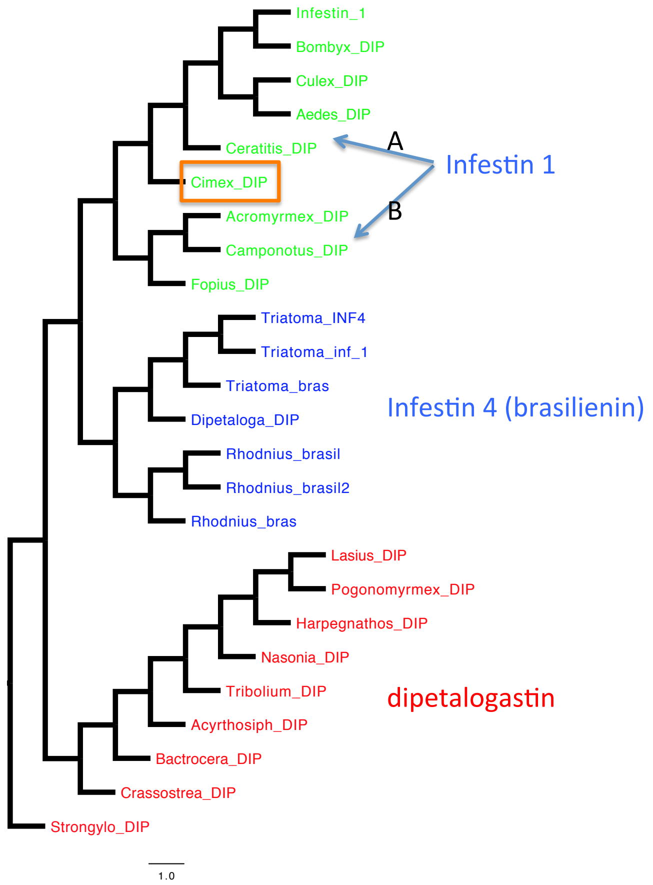

Supplementary Figure 11. Phylogenetic tree of insect infestins. The tree was generated using maximum parsimony and with a Strongylocentrotus infestin as an outgroup. Random Additions (n=100) were used with Tree Bisection Reconnection (TBR) branch swapping to obtain the tree. The colored names in the tree refer to the three major kinds of infestins that are suggested by this analysis. Red indicates the dipetalogastin family, the blue indicates the brasiliensin family (or infestin 4) and the green represents the infestin 1 family. The Cimex infestin is in the orange square.

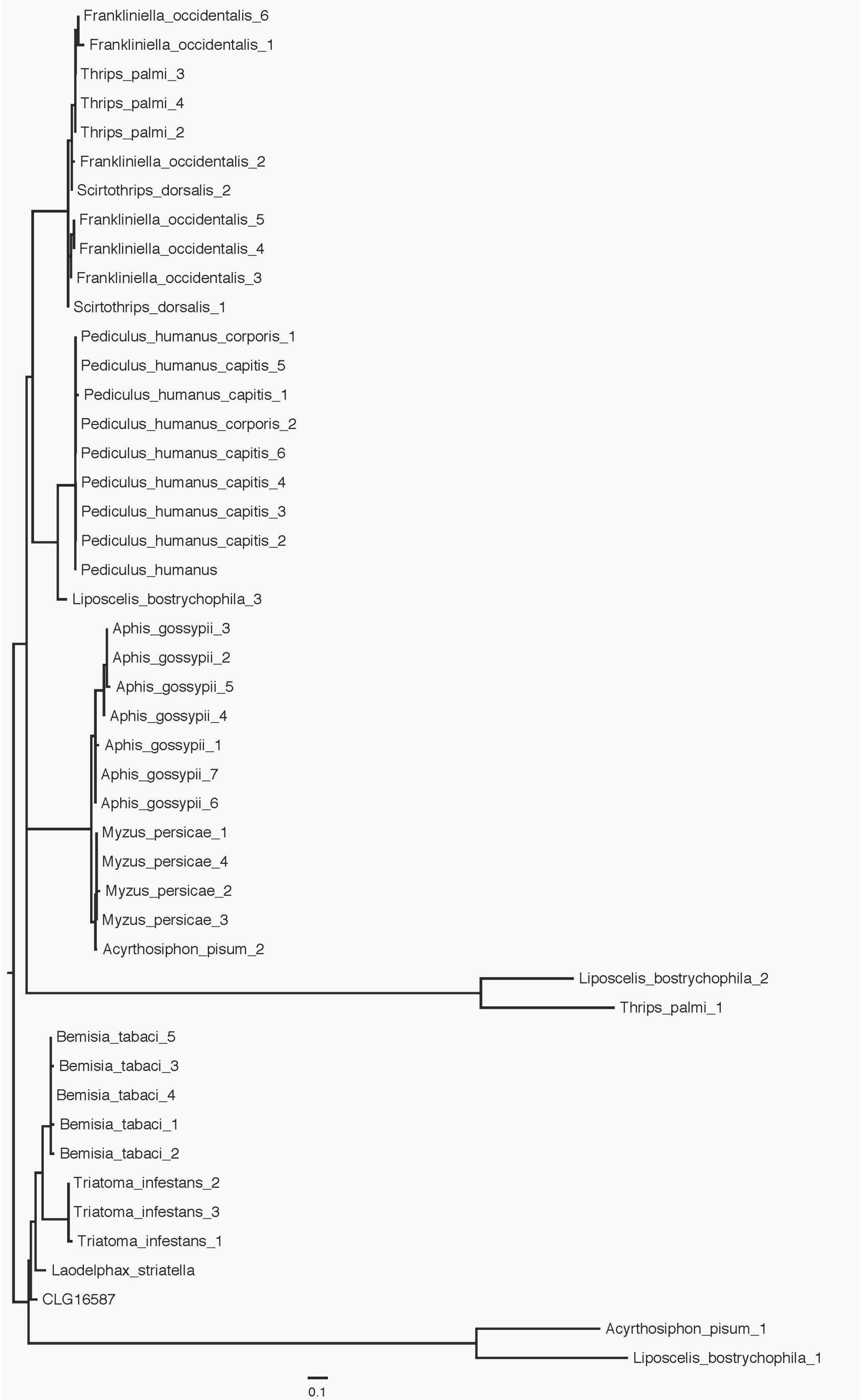

Supplementary Figure 12. Voltage-gated sodium channel gene tree. Maximum-likelihood tree showing the *Cimex lectularius* gene (CLG16587) clustering with other hemipteran homologs.

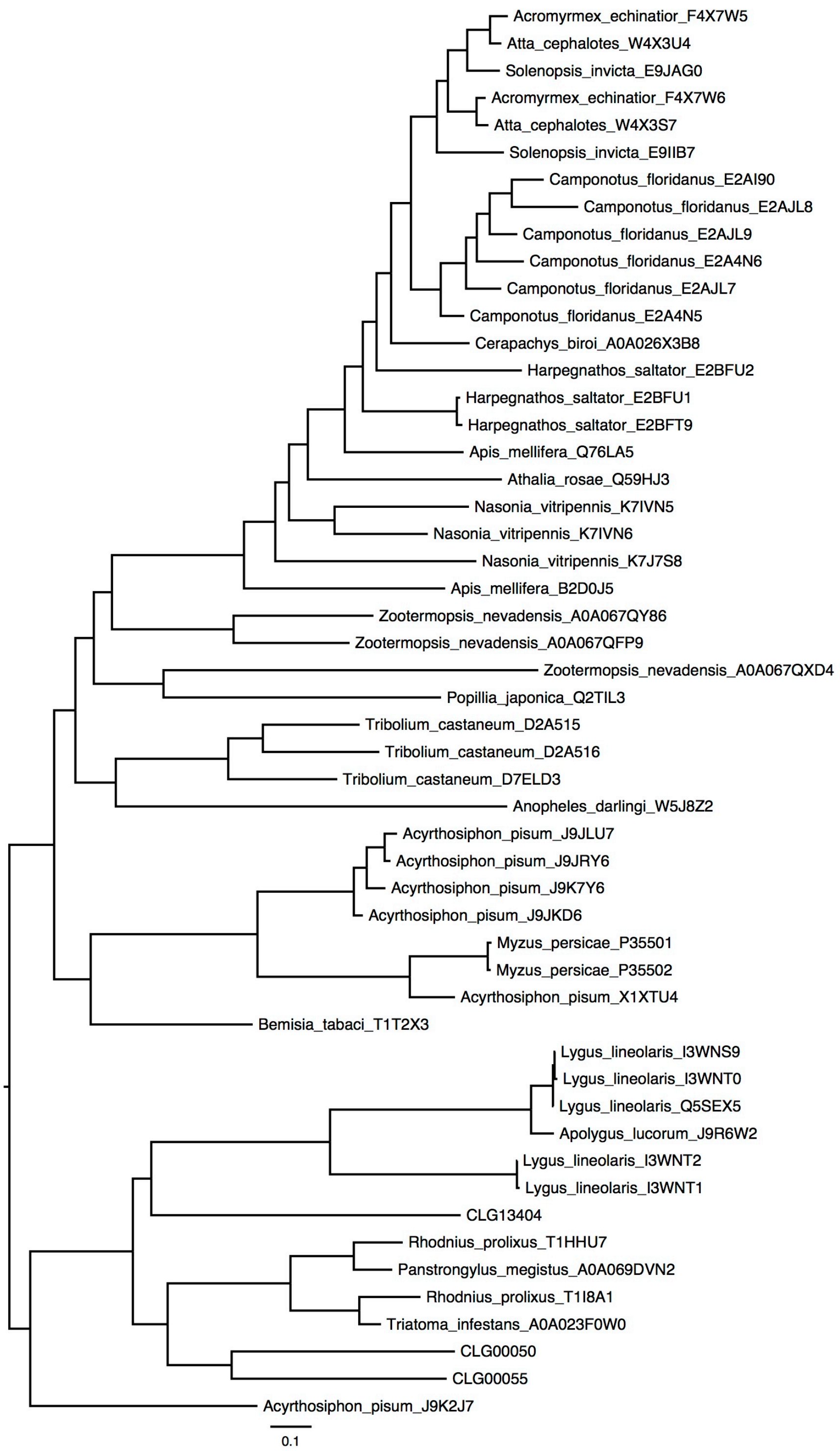

Supplementary Figure 13. A phylogenetic tree of the bedbug and other insect esterase genes. Three bedbug homologs (CLG00050, CLG13404, CLG00055) were found in bedbug with partial identity to other blood-feeding hemipteran insects (kissing bugs, *R. prolixus* and *T. infestans*), nested within the main cimicomorph esterase clade. Same maximum liklihood scale as Supplemental Supplementary Figure 12.

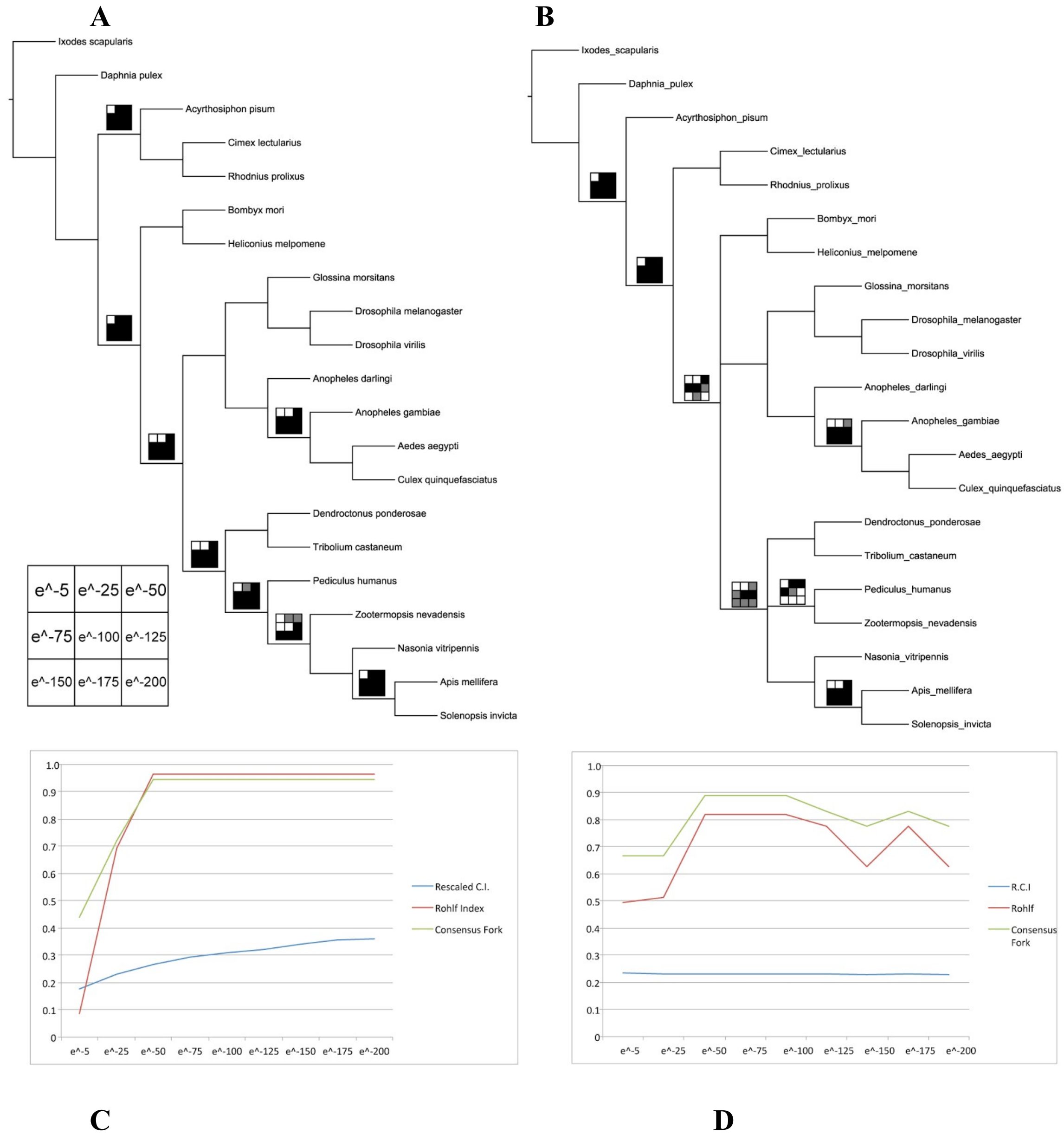

Supplementary Figure 14. Evolutionary relationships based on gene presence-absence. Panels (A) maximum parsimony and (B) maximum likelihood show the dynamics of E-value cutoff on the consistency of phylogenetic trees generated using the gene presence-absence information. Majority-rule consensus parsimony and likelihood trees were calculated (bootstrap = 10,000), and for both of the majority-rule topologies, the relative support of each gene family matrix is shown as "Navajo rugs" [3] at each node. Black boxes indicate nodal agreement, white boxes indicate disagreement, and gray boxes indicate agreement with bootstrap support > 70%. To measure character consistency, the Rescaled Consistency Index (RCI) [4] was computed. To measure nodal agreement, the Consensus Fork Index (CFI) [5] and Rohlf consensus index 1 [6] were computed. The graphs (panels C and D) examine the dynamics of E-value cutoff analyses. These figures demonstrate an optimal E-value cutoff in the range e-50–e-75 for this dataset. All nodes on this tree received 100% bootstrap support.

Supplementary Tables

Supplemental Table 1 - Illumina libraries used in transcriptome assembly.

Source	Read type	Replicate 1	Replicate 2	Replicate 3
Adult	PE100	515376920 x 2	NA	NA
1st Instar	SE50	4,886,399	6,313,424	5,778,247
2nd Instar	SE50	4,591,633	5,982,408	4,670,338
3rd Instar	SE50	3,817,205	5,241,204	5,576,136
4th Instar	SE50	4,468,666	5,267,733	4,620,707
5th Instar	SE50	4,626,900	5,659,537	5,480,717
Adult Female	SE50	4,920,227	5,730,096	6,032,540
Adult Male	SE50	4,966,525	6,787,451	4,972,552

Note (PE100, 100bp paired-end reads; SE50, 50bp single-end reads)

Supplemental Table 2 - Description of Illumina paired-end libraries used in genome assembly.

Insert Length	Read Pairs	Total Mb	Covearge
185 bp	119,416,422	23,883	34.27
367 bp	42,382,611	8,477	12.16
3000 bp	24,605,824	4,921	7.06
6000 bp	65,111,047	13,022	18.68

Supplemental Table 3 - Moleculo Base-level accuracy

Library	Moleculo 1	Moleculo 2	Moleculo 3
Length	< 7501 bp	7501-9000 bp	> 9000 bp
Aligned Bases	542,975,405	340,477,235	213,340,013
Total Edit Distance	2,179,239	7,247,951	7,718,261
Percent Identity	99.60%	97.90%	96.40%

Supplemental Table 4 - Genome Assembly Statistics

Parameter	ALLPATHS-LG	Metaassembler
minimum contig size for reporting	1,000	1,000
number of scaffolds	13,151	12,259
total scaffold length, with gaps	713,608,678	697,867,761
N50 scaffold size in kb, with gaps	947	971
N95 scaffold size in kb, with gaps	8,643	9,736
maximum scaffold size	6,361,674	6,212,894

Supplemental Table 5- Comparison of differential gene expression between bedbug developmental stages.

Comparison	Differentially expressed genes	Upregulated genes	Downregulated genes
1__vs__2	4386	2467	1919
1__vs__3	1983	572	1411
1__vs__4	1307	96	1211
1__vs__5	627	8	619
1__vs__Female	2755	742	2013
1__vs__Male	791	484	307
2__vs__3	120	91	29
2__vs__4	537	303	234
2__vs__5	2191	1230	961
2__vs__Female	5990	1816	4174
2__vs__Male	6242	2715	3527
3__vs__4	18	13	5
3__vs__5	588	552	36
3__vs__Female	3192	1358	1834
3__vs__Male	3524	2241	1283
4__vs__5	421	416	5
4__vs__Female	1908	1034	874
4__vs__Male	2590	2004	586
5__vs__Female	3267	1367	1900
5__vs__Male	2435	1777	658
Female__vs__Male	2886	2067	819

Supplemental Table 6: Repetitive Element Classification

Category	Number of Elements	Number of BP covered	Percentage of Genome
**SINEs:**	140178	17327911	2.5%
ALUs	0	0	0.0%
MIRs	1482	99463	0.0%
**LINEs:**	331801	79880695	11.5%
LINE1	103	6461	0.0%
LINE2	9892	3429637	0.5%
L3/CR1	6887	1707471	0.3%
**LTR elements:**	50201	6852704	1.0%
ERVL	8	477	0.0%
ERVL-MaLRs	5	239	0.0%
ERV_classI	190	32207	0.0%
ERV_classII	6	333	0.0%
**DNA elements:**	104574	27677734	4.0%
hAT-Charlie	4389	1283082	0.2%
TcMar-Tigger	904	199019	0.0%
**Unclassified:**	373611	64684425	9.3%
Total interspersed Repeats	196423469	196423469	28.2%
Small RNA:	62012	5297714	0.8%
Satellites:	1	67	0.0%
Simple repeats:	341954	18158644	2.6%
Low complexity:	78295	5919438	0.8%

Supplemental Table 7 - Numbers of Matches of Cimex genes to each microbial genus from TBLASTX

Genus	Number of Matches
Wolbachia	114
Clostridium	25
Cyanothece	24

Bacillus	22
Thermococcus	20
Myxococcus	20
Sorangium	17
Burkholderia	15
Pyrococcus	14
Oscillatoria	14
Pectobacterium	13
Spirochaeta	12
Pseudomonas	12
Legionella	12
Geobacter	12
Archaeoglobus	12
Thermofilum	11
Methanobacterium	11
Vibrio	10
Streptomyces	10
Paenibacillus	10
Methanocaldococcus	10
Desulfovibrio	10
Aciduliprofundum	10
Rhodothermus	9
Rhodospirillum	9
Nostoc	9
Magnetospirillum	9
Leptospira	9
Dickeya	9
Desulfatibacillum	9
Caldilinea	9
Synechococcus	8
Sulfolobus	8
Serratia	8
Planctomyces	8
Methanopyrus	8
Deinococcus	8
Anabaena	8
Xenorhabdus	7
Shewanella	7
Rhizobium	7
Pleurocapsa	7
Photorhabdus	7
Opitutus	7
Mycobacterium	7
Hyphomicrobium	7

Azospirillum	7
Trichodesmium	6
Stigmatella	6
Ralstonia	6
Pyrobaculum	6
Methanosaeta	6
Lactobacillus	6
Haliangium	6
Enterobacter	6
Desulfotomaculum	6
Corallococcus	6
Calothrix	6
Bacteroides	6
Agrobacterium	6
Thermodesulfatator	5
Staphylococcus	5
Saprospira	5
Rubrobacter	5
Rivularia	5
Providencia	5
Pelobacter	5
Micavibrio	5
Methylobacterium	5
Methanotorris	5
Methanocella	5
Marivirga	5
Ignavibacterium	5
Hyperthermus	5
Herpetosiphon	5
Francisella	5
Desulfomonile	5
Cyanobacterium	5
Bdellovibrio	5
Bacteriovorax	5
Arthrospira	5
Anaeromyxobacter	5
Alkaliphilus	5
Waddlia	4
Thermoanaerobacter	4
Sulfobacillus	4
Streptococcus	4
Staphylothermus	4
Sphaerobacter	4
Runella	4

Rhodococcus	4
Psychromonas	4
Parachlamydia	4
Nitrosomonas	4
Methanothermus	4
Methanothermobacter	4
Methanosarcina	4
Methanococcus	4
Metallosphaera	4
Mesorhizobium	4
Marinomonas	4
Marinobacter	4
Helicobacter	4
Gloeobacter	4
Dictyoglomus	4
Desulfobacterium	4
Cylindrospermum	4
Cyclobacterium	4
Crinalium	4
Chlorobium	4
Brevibacillus	4
Amycolatopsis	4
Acaryochloris	4
Zunongwangia	3
Zobellia	3
Turneriella	3
Truepera	3
Tistrella	3
Thermotoga	3
Terriglobus	3
Streptosporangium	3
Stenotrophomonas	3
Stanieria	3
Sodalis	3
Singulisphaera	3
Rickettsia	3
Rhodopirellula	3
Rahnella	3
Pyrolobus	3
Pseudoalteromonas	3
Proteus	3
Pirellula	3
Pedobacter	3
Parvibaculum	3

Owenweeksia	3
Nitratifractor	3
Methylomonas	3
Methanoplanus	3
Methanomassiliicoccus	3
Methanolobus	3
Methanohalophilus	3
Methanohalobium	3
Magnetococcus	3
Leptolyngbya	3
Gluconobacter	3
Glaciecola	3
Frankia	3
Exiguobacterium	3
Desulfurococcus	3
Desulfosporosinus	3
Cytophaga	3
Cupriavidus	3
Coxiella	3
Colwellia	3
Chloroflexus	3
Chitinophaga	3
Chamaesiphon	3
Cenarchaeum	3
Caldivirga	3
Caldisphaera	3
Caldisericum	3
Caldicellulosiruptor	3
Bradyrhizobium	3
Blattabacterium	3
Actinoplanes	3
Achromobacter	3
Zymomonas	2
Xanthomonas	2
Vulcanisaeta	2
Tsukamurella	2
Treponema	2
Thioalkalivibrio	2
Thermomicrobium	2
Thermobaculum	2
Teredinibacter	2
Syntrophobacter	2
Synergistetes	2
Sulfurihydrogenibium	2

Spirosoma	2
Sinorhizobium	2
Simiduia	2
Rothia	2
Roseobacter	2
Roseburia	2
Rhodopseudomonas	2
Pseudovibrio	2
Propionibacterium	2
Porphyromonas	2
Polaribacter	2
Plautia	2
Phycisphaera	2
Photobacterium	2
Pelotomaculum	2
Pelodictyon	2
Pantoea	2
Paludibacter	2
Octadecabacter	2
Nitrosopumilus	2
Niastella	2
Mycoplasma	2
Morganella	2
Microcystis	2
Microcoleus	2
Methylocystis	2
Methanobrevibacter	2
Melioribacter	2
Marinitoga	2
Leptospirillum	2
Leifsonia	2
Klebsiella	2
Kangiella	2
Isosphaera	2
Ignisphaera	2
Ignicoccus	2
Hydrogenobacter	2
Hirschia	2
Herbaspirillum	2
Halothermothrix	2
Haloterrigena	2
Halorhabdus	2
Haloferax	2
Haliscomenobacter	2

Halanaerobium	2
Hahella	2
Gramella	2
Geobacillus	2
Flexibacter	2
Fervidicoccus	2
Ferroglobus	2
Enterococcus	2
Emticicia	2
Echinicola	2
Desulfurivibrio	2
Desulfomicrobium	2
Desulfohalobium	2
Desulfococcus	2
Desulfitobacterium	2
Dactylococcopsis	2
Cronobacter	2
Comamonas	2
Chroococcidiopsis	2
Chloroherpeton	2
Cellulomonas	2
Cardinium	2
Calditerrivibrio	2
Belliella	2
Bartonella	2
Azoarcus	2
Amphibacillus	2
Alteromonas	2
Alkalilimnicola	2
Acidovorax	2
Acidithiobacillus	2
Acidimicrobidae	2
Acidilobus	2
Acetohalobium	2
Weeksella	1
Verrucosispora	1
Veillonella	1
Variovorax	1
Thioflavicoccus	1
Thiocystis	1
Thermosphaera	1
Thermosediminibacter	1
Thermoproteus	1
Thermoplasma	1

Thermomonospora	1
Thermogladius	1
Thermodesulfovibrio	1
Thermocrinis	1
Thermoanaerobacterium	1
Thermincola	1
Thermaerobacter	1
Thauera	1
Tannerella	1
Synechocystis	1
Symbiobacterium	1
Sulfurovum	1
Sulfuricurvum	1
Strawberry	1
Starkeya	1
Stackebrandtia	1
Spiroplasma	1
Sphingomonas	1
Solitalea	1
Simkania	1
Sideroxydans	1
Salinispora	1
Salinibacter	1
Salinarchaeum	1
Saccharothrix	1
Ruminococcus	1
Rubrivivax	1
Roseiflexus	1
Rhodanobacter	1
Ramlibacter	1
Psychroflexus	1
Pseudonocardia	1
Pseudanabaena	1
Prosthecochloris	1
Prochlorococcus	1
Prevotella	1
Polynucleobacter	1
Phenylobacterium	1
Phaeobacter	1
Persephonella	1
Paracoccus	1
Orientia	1
Onion	1
Oceanithermus	1

Oceanimonas	1
Novosphingobium	1
Nonlabens	1
Nocardia	1
Nitrosospira	1
Nitrobacter	1
Nautilia	1
Muricauda	1
Moraxella	1
Modestobacter	1
Methylotenera	1
Methylomicrobium	1
Methylococcus	1
Methylocella	1
Methylacidiphilum	1
Methanothermococcus	1
Methanosphaerula	1
Methanosphaera	1
Methanosalsum	1
Methanoregula	1
Methanomethylovorans	1
Methanoculleus	1
Mesoplasma	1
Melissococcus	1
Meiothermus	1
Marinithermus	1
Maricaulis	1
Maribacter	1
Mannheimia	1
Mahella	1
Macrococcus	1
Listeria	1
Leptotrichia	1
Leptothrix	1
Leisingera	1
Leadbetterella	1
Lactococcus	1
Lacinutrix	1
Kribbella	1
Kitasatospora	1
Idiomarina	1
Hydrogenobaculum	1
Hippea	1
Heliobacterium	1

Halovivax	1
Halothiobacillus	1
Halothece	1
Halorubrum	1
Haloquadratum	1
Halopiger	1
Halophilic	1
Halogeometricum	1
Halobacteroides	1
Halalkalicoccus	1
Haemophilus	1
Granulicella	1
Granulibacter	1
Gloeocapsa	1
Geitlerinema	1
Fusobacterium	1
Frateuria	1
Fluviicola	1
Flexistipes	1
Flavobacterium	1
Flavobacteriaceae	1
Fibrella	1
Fervidobacterium	1
Ferrimonas	1
Faecalibacterium	1
Eubacterium	1
Escherichia	1
Elusimicrobium	1
Eggerthella	1
Edwardsiella	1
Dyadobacter	1
Desulfurispirillum	1
Desulfotalea	1
Desulfocapsa	1
Desulfarculus	1
Denitrovibrio	1
Delftia	1
Dehalogenimonas	1
Deferribacter	1
Dechloromonas	1
Conexibacter	1
Comamonadaceae	1
Clavibacter	1
Citrobacter	1

Chromobacterium	1
Chlamydia	1
Cellvibrio	1
Cellulophaga	1
Caulobacter	1
Catenulispora	1
Carnobacterium	1
Carboxydothermus	1
Candidate	1
Butyrivibrio	1
Butyrate-producing	1
Brucella	1
Brevundimonas	1
Bibersteinia	1
Beijerinckia	1
Azorhizobium	1
Arthrobacter	1
Aromatoleum	1
Anoxybacillus	1
Anaerolinea	1
Amycolicicoccus	1
Ammonifex	1
Alcanivorax	1
Akkermansia	1
Agromonas	1
Aggregatibacter	1
Aeropyrum	1
Aeromonas	1
Aequorivita	1
Actinosynnema	1
Actinobacillus	1
Acidothermus	1
Acidobacterium	1
Acidianus	1
Acidaminococcus	1

Genes found to be microbial by Alien_Index

CLG18395
CLG30550
CLG27621
CLG07002
CLG37794
CLG34355

CLG19415
CLG20121
CLG36171
CLG36804
CLG22368
CLG04851
CLG28628
CLG31459
CLG00153
CLG24980
CLG36172
CLG20119
CLG04852
CLG27458
CLG18396
CLG02682
CLG25156
CLG25533
CLG22534
CLG02677
CLG25532
CLG21625
CLG37795
CLG29893
CLG36170
CLG02689
CLG22538
CLG34352
CLG24982
CLG22536
CLG02678
CLG04850
CLG19414
CLG08570
CLG01871
CLG24984
CLG02684
CLG29977
CLG25534
CLG18444
CLG13330
CLG36168
CLG26542
CLG30551
CLG22535

CLG04849
CLG26064
CLG22369
CLG30549
CLG20117
CLG13405
CLG31549
CLG24157
CLG29532
CLG24981
CLG33576
CLG17711
CLG36605
CLG22370
CLG18394
CLG24979
CLG30232
CLG29759
CLG25157
CLG18393
CLG24995
CLG07151
CLG36806
CLG22373
CLG06192
CLG02688
CLG08100
CLG36174
CLG10509
CLG00154
CLG34109
CLG20118
CLG22371
CLG32732
CLG20122
CLG18392
CLG36805
CLG22537
CLG02679
CLG02690
CLG09293
CLG34357
CLG02687
CLG24996
CLG22593

CLG34354
CLG27186
CLG02683
CLG02685
CLG02676
CLG20120
CLG02680
CLG00156
CLG24983
CLG34353
CLG36176
CLG18391
CLG22980
CLG37793
CLG13329
CLG27622
CLG36175
CLG03486

Supplemental Table 8 Three member hydrogen network between residues 96-98-168.
X-ray structures used as templates for homology models highlighted in green.

PDB code	96	98	168	Network
2FB9	D	D	K	yes
2YZG	D	D	K	yes
2YZM	D	D	K	yes
2YZN	D	D	K	yes
2ZDG	D	D	K	yes
2ZDH	D	D	K	yes
2ZDQ	D	D	K	yes
3E5N	D	D	K	yes
3I12	D	D	K	yes
3LWB	D	E	K	yes
3Q1K	D	D	K	yes
3R5F	D	D	K	yes
3RFC	D	D	K	yes
4L1K	D	D	K	yes
4ME6	D	D	K	yes
1E4E	D	S	K	yes
3TQT	E	D	R	yes

1EHI	D	A	K	yes
2I80	D	L	K	yes
2I87	D	L	K	yes
2I8C	D	L	K	yes
3N8D	D	L	K	yes
3R5X	D	L	E	no
4C5A	D	L	E	no
4C5B	D	L	E	no
4C5C	D	L	E	no
4FU0	D	L	E	no
**1I0V**	**D**	**L**	**E**	**no**
1IOW	D	L	E	no
2DLN	D	L	E	no
2PVP	D	L	E	no
3R23	D	L	E	no

Supplemental Table 9 - Anticoagulants and Bloodmeal-related DEGs

Query	SP	e-value	Acc	Definition
				**Apyrase**
CLG18094	+	1.00E-99	CAE46445	79 kDa salivary apyrase precursor [Triatoma infestans]
				**Salivary inositol polyphosphate 5-phosphatase**
CLG02551	++	4.00E-62	AAB08434	salivary inositol polyphosphate 5-phosphatase [Rhodnius prolixus]
CLG36692	++	1.00E-31	AAB08434	salivary inositol polyphosphate 5-phosphatase [Rhodnius prolixus]
CLG14908	++	7.00E-61	AAB08434	salivary inositol polyphosphate 5-phosphatase [Rhodnius prolixus]
CLG18721	++	6.00E-60	AAB08434	salivary inositol polyphosphate 5-phosphatase [Rhodnius prolixus]
				**Infestin**
CLG11091	++	6.00E-09	AAK57342	thrombin inhibitor infestin precursor [Triatoma infestans]
CLG11092	+	6.00E-09	AAK57342	thrombin inhibitor infestin precursor [Triatoma infestans]
CLG14478	++	5.00E-20	AAK57342	thrombin inhibitor infestin precursor [Triatoma infestans]
				**Serine Proteases**
CLG29395	++	0	BAN20353	prolylcarboxypeptidase, putative [Riptortus pedestris]
CLG00735	++	4.00E-174	EDS34712	serine protease [Culex quinquefasciatus]
CLG09902	+	1.00E-13	EFN87035	serine protease snake [Harpegnathos saltator]
CLG34389	++	7.00E-53	ETN60567	serine protease [Anopheles darlingi]
CLG33858	++	8.00E-104	ETN60567	serine protease [Anopheles darlingi]

CLG20224	++	4.00E-13	NP_001155164	venom protein R precursor [Nasonia vitripennis]
CLG15203	++	7.00E-17	NP_001155164	venom protein R precursor [Nasonia vitripennis]
				**Other Salivary**
CLG20238	++	2.00E-24	ABR27888	putative salivary secreted protein [Triatoma infestans]
CLG20224	++	3.00E-21	ABR27888	putative salivary secreted protein [Triatoma infestans]
CLG15203	++	1.00E-27	ABR27888	putative salivary secreted protein [Triatoma infestans]
CLG32648	++	1.00E-14	ABR27836	salivary secreted protein [Triatoma infestans]
CLG20227	++	7.00E-18	ABR27888	putative salivary secreted protein [Triatoma infestans]
				**Other Secreted**
CLG02599	++	6.00E-10	ACH56920	salivary lysozyme [Simulium vittatum]
CLG19605	++	2.00E-153	EAT38110	lipocalin-1 interacting membrane receptor (limr) [Aedes aegypti]
CLG37461	++	0	EAT39655	metalloprotease m41 ftsh [Aedes aegypti]
CLG00050	++	1.00E-138	EZA62484	Venom carboxylesterase-6 [Cerapachys biroi]
CLG24957	+	2.00E-07	ABG01864	putative accessory gland protein [Gryllus veletis] (gryllus gland)
CLG10344	++	3.00E-40	ABR27829	salivary trypsin [Triatoma infestans]
CLG21399	++	4.00E-08	AAF28384	lung surfactant protein A [Sus scrofa]
CLG04610	+	3.00E-09	EU045345	50 kDa midgut protein [Phlebotomus papatasi]

Supplemental Table 10 - Trinity transcriptome assembly statistics.

Parameter	Size
N50	3,550
N95	341
Mean contig size (min–max)	1,596.60
Assembly size (bp)	216,321,741
No. of sequences	135,489

Supplementary Methods

Raw sequence data

The genome assembly validated by the National Center for Biotechnology Information (NCBI), where it was checked for adaptors, primers, gaps, and low-complexity regions. The genome assembly has been approved and given the accession number JRLE00000000 and BioProject

PRJNA259363. All genome sequencing data has been deposited in the Sequence Read Archive (SRA) with accession number SRS749263. RNA-seq data is available as FASTQ files and were quality-checked and deposited in the SRA with accession SRR1790655.

Biological samples

The bedbugs were taken from a Harlan strain colony maintained by Louis Sorkin (American Museum of Natural History). The Har-73 strain was originally collected by Harold Harlan in 1973 from an infestation at the U.S. Army barracks in Fort Dix, NJ, and has been raised as a laboratory pesticide-susceptible strain since that time.

Bedbug collection and feeding

Bedbugs were reared in ~236.6 ml (8 fl oz) glass canning jars where the metal covers had a 250-350 µm hole mesh screening heat-glued on the inside. Heat glue was applied to the outer circumference of the screen surface to leave a 3 cm diameter central circle of exposed screen. Folded cardboard was used as substrate. Jars were inverted on a human arm for feeding for 30 min on a monthly basis. Jars were kept in plastic box with an open lid and left at room temperature. Specimens used for nucleic acids extraction were 1st instar nymphs that recently hatched but had not taken any blood meals (~1 mm in length, pale to white in color).

DNA & RNA isolation

High molecular weight DNA (>10kb as visualized through agarose gel electrophoresis) was isolated from ~30 1st instar nymphs using the DNeasy Blood & Tissue kit (QIAGEN). Total RNA was isolated from ~30 individuals for each nymph stage and ~5 individuals for each adult sex. The RNA extractions were performed using a Trizol / RNeasy (QIAGEN) hybrid protocol, as detailed in [1].

High throughput sequencing library quality check

Moleculo sequences were segregated into 3 bins by length: short (<7,501 bp), medium (7,501-9,000 bp), and long (>9000 bp). There were 53,5541 short, 30,150 medium, and 6,216 long sequences. The long reads were used to confirm the insert length of the overlapping fragment libraries (185 bp insert) by aligning (using BWA [7]) a single lane of the reads to all Moleculo reads >9000 bp. There were a total of 6,216 such sequences. The insert length of pairs where both pairs mapped was calculated. A sample of 6,926,206 HiSeq reads were randomly selected and trimmed using SolexaQA (http://solexaqa.sourceforge.net) using a quality value filter of Q30. Each set of Moleculo sequences was indexed using BWA v0.7.5a (http://bio-bwa.sourceforge.net). Alignments of the filtered HiSeq data to each Moleculo dataset were performed using the “mem” algorithm of BWA with 30 threads and standard settings. Alignments were extracted in BAM format using samtools (http://www.htslib.org) with -F set to 4. The ‘MD’ tag was added to the resulting BAM files using the calmd command of samtools producing SAM files containing this tag. The MD tag allowed for two pieces of information to be extracted from the alignments: the total number of nucleotides included in each alignment and the edit distance between the query and reference sequences. The command used for obtaining the total sequence alignment length was

```
cat sample_seqs.aln.md.sam | awk '{print $10"\t"$12}' | awk -F: '{print $1"\t"$2"\t"$3}' |
awk '{print length($1)}' |  paste -sd+ | bc > sample_seqs.aln.seq_length
```

Edit distance for each alignment was obtained using the following command:

```
grep -o "NM:i:.*\s" sample_seqs.aln.md.sam | awk -F: '{print $3}' | awk '{print $1}' | paste
-sd+ | bc &> sample_seqs.aln.tot_distance
```

The percentage identity between the sequences was obtained by dividing the total edit distance by the total alignment sequence length and converting the value to a percentage.

Insert Size Validation

Insert sizes of the DNA paired-read sequencing libraries were validated using an assembly and alignment strategy. First, reads were trimmed for adapters using SeqPrep (https://github.com/jstjohn/SeqPrep). Adapters were specified as follows: -A AGATCGGAAGAGCACACGTCTGAACTCCAGTCAC -B AGATCGGAAGAGCGTCGTGTAGGGAAAGAGTGTA. The remaining reads were then quality trimmed using SolexaQA using a phred score cutoff of 20 (-h 20) for DynamicTrim.pl and a minimum trimmed read length of 23 (-l 23) for LengthSort.pl. Reads were then error trimmed using the ErrorCorrectReads.pl command in ALLPATHS-LG v44431 (http://www.broadinstitute.org/software/allpaths-lg/blog). The parameters used for all reads were PHRED_ENCODING=33 and THREADS=10 and the parameter MAX_MEMORY_GB ranged between 20 and 50.

The ABySS [8] assembly program was used to assemble the trimmed sequence reads.

Alignments of the fragment library were performed to the longest set of Moleculo reads using BWA using default options except for the multiple core option -t 30. The resulting SAM file was converted into a BAM file using samtools with the view command and -bS option. Insert sizes were extracted from the resulting BAM file using samtools options view, -F 12 -f 67 and a one-line Perl script:

```
perl -lane 'if (abs($F[8])<1000 && abs($F[8])>0){print abs($F[8])}'
```

The resulting file of insert sizes were plotted using the Python library matplotlib and descriptive statistics were generated using the Python library scipy.

Genome assembly

The genome assembly validated by the National Center for Biotechnology Information (NCBI), where it was checked for adaptors, primers, gaps, and low-complexity regions.

ABySS

In order to provide accurate insert sizes for the ALLPATHS-LG assembly, an initial ABySS assembly was generated *de novo*. ABySS 1.5[2] provides the ability to specify paired-end (PE) read libraries to the assembly without specifying the expected insert size parameters. This allowed for the use of PE information in the assembly. Assemblies were produced for a range of *k*-mer lengths: 23, 33, 43, 53, and 63. Overlapping PE reads for fragment libraries were aligned to each of the ABySS assemblies to ensure consistency across *k*-mer values. Insert sizes for reads for which both pairs mapped were calculated using samtools.

ALLPATHS-LG

The genome assembly was performed using ALLPATHS-LG R44837 (http://www.broadinstitute.org/software/allpaths-lg/blog). The assembly used default settings with the exception of the minimum contig size being set to 200bp (MIN_CONTIG=200) and 20 threads (THREADS=20) when running the RunAllPathsLG command. Four sequencing libraries were provided for the assembly: a fragment library with a mean insert size of 160 bp and SD=20 bp, a jumping library with a mean insert size of 600bp and SD=150 bp, a jumping library with a mean insert size of 2,100 bp and SD=500 bp, and a jumping library with a mean insert size of 3,700 bp and SD=500 bp.

Moleculo

A total of 571,913 Moleculo reads were generated, ranging in size from 1500 to 18,740 bp (mean=3,481±1,923 bp), representing a total of ~4× coverage of the genome (1.99 Gb total sequence production). The reads were assembled using the Celera Assembler v8.0 (http://wgs-assembler.sourceforge.net) with the bogart unitig algorithmic implementation [3]. Note that this version of the assembler has been enhanced to support reads as long as 32 kb so to accommodate Moleculo and other long-read sequencing technologies. All other parameters were set to their recommended values. In light of the low coverage, the assembler created 59,785 contigs spanning 473,254,128 bp with an N50 size of 10,674 bp (max=193,467 bp). Because no mate-pairs were used in the assembly, no scaffolds were available from these data.

Metassembler

The ALLPATHS and Moleculo assemblies were combined into a single assembly using Metassembler 1.1 [4] with the following parameters: bowtie2_threads=24, bowtie2_maxins=2424, bowtie2_minins=5024, mateAn_A=3074, mateAn_B=4374. The ALLPATHS assembly was set as the primary assembly with the Moleculo assembly being secondary. In order to keep ALLPATHS scaffolds which do not have alignments in the Moleculo assembly, the following parameters were used meta2fasta_do=1, meta2fasta_keepFlag=0, meta2fasta_sizeFilterP=200. This approach was taken due to the large amount of missing sequence in the Moleculo assembly.

BioNano genome mapping

High-molecular weight DNA extraction

High-molecular weight (HMW) DNA extraction was performed based on the protocols from Zhang et al. (2011). Bedbug embryos were rinsed in 0.7% NaCl and then soaked in 50% bleach. After being rinsed again, they were washed with Mosquito Buffer (MB) (100 mM NaCl, 200 mM sucrose, 10 mM EDTA (pH 9.4), and 7.5 µL BME) and diced with a razor blade until pulp. They were ground gently with a pestle in a microcentrifuge tube and then allowed to settle for 2 min. The supernatant was transferred to a new tube. Two hundred µL of MB was added to the remaining insoluble material and grinding was repeated, followed by settling and removing the supernatant and combining it with the first supernatant. This was repeated until the supernatant was clear (~3 additional times). The whole supernatant was passed through a 40-µM filter and then centrifuged for 5 minutes at 4000 × g. The supernatant was discarded and the pellet was washed with PBS 2×. The pellet was finally resuspended in 40 µL of cell resuspension buffer and gel plugs were made as recommended for the CHEF Mammalian Genomic DNA Plug Kit (BioRad cat. No. 170-3591). Plugs were incubated with lysis buffer and proteinase K for 4 h at 50°C. After a wash, 2.5mL RNase Buffer (10mM Tris (pH 7.5) and 15mM NaCl) were added, followed by addition of 50 µL RNaseA (QIAGEN). The plugs were washed and then solubilized with GELase (Epicentre). The

purified DNA was subjected to 4 h of drop dialysis (Millipore cat. No. VCWP04700) and quantified on a Nanodrop 1000 spectrophotometer (Thermo Scientific) and/or the Quant-iT dsDNA Assay Kit (Molecular Probes, Life Technologies).

DNA labeling

DNA was labeled according to commercial protocols using the IrysPrep Reagent Kit (BioNano Genomics, Inc). Specifically, 300 ng of purified genomic DNA was nicked with 4 U of nicking endonuclease Nt.BspQI and 3 U of Nt.BbvCI or Nb.BbvCI (New England BioLabs) at 37°C for 2 h in NEB Buffer 3. The nicked DNA was labeled with a fluorescent-dUTP nucleotide analog using Taq polymerase (New England BioLabs) for 1 h at 72°C. After labeling, the nicks were ligated with Taq ligase (New England BioLabs) in the presence of dNTPs. The backbone of fluorescently labeled DNA was stained with YOYO-1 Iodide (Molecular Probes, Life Technologies).

Data collection

The DNA was loaded onto the nanochannel array of BioNano Genomics IrysChip using electrophoresis. Linearized DNA molecules were then imaged and repeated cycles of DNA loading and imaging using the BioNano Genomics Irys system was performed. The DNA molecule backbones (YOYO-1 stained) and locations of fluorescent labels along each molecule were detected using the software package IrysView (http://www.bionanogenomics.com/products/irysview). The label locations of each DNA molecule were reported to produce an individual single-molecule map.

Single-molecule alignment against sequence assembly

In-silico maps were generated based on the sequence assembly scaffold for alignment against single-molecule maps. Single-molecule maps were aligned to the in-silico maps using software tools developed at BioNano Genomics. Alignments were obtained using a dynamic programming approach maximizing the scoring function that represented the likelihood of a pair of intervals being similar (Anantharaman TS, 2001). The likelihood scores were calculated based on a noise model which took into account fixed sizing error, sizing error which scales linearly with the interval size, misaligned sites (false positives and false negatives), and optical resolution. An alignment *P*-value threshold of 1e-9 was used to minimize false positive alignments.

Transcriptome assembly

The bedbug transcriptome was produced using the Trinity assembler r2012-10-05 [7]. In order to reduce the amount of redundant information fed to Trinity, duplicate sequences among the 631,227,170 50-bp single-end reads were removed using the fastq-mcf program from the ea-utils library. This was achieved using the command line options -0 -D 50 n/a. Prior to assembly, the adapter sequencers were trimmed from all reads using SeqPrep v1.0 [8] with the following parameters: -A AGATCGGAAGAGCACACGTCTGAACTCCAGTCAC -B AGATCGGAAGAGCGTCGTGTAGGGAAAGAGTGTA. Basecall quality trimming was then performed using SolexaQA [9] with a phred score cutoff of 20 (-h 20) in DynamicTrim.pl and a minimum trimmed read length of 23 (-l 23) in LengthSort.pl. Trinity was run with the following parameters: --seqType fq --JM 200G --CPU 32. The assembly statistics are shown in **Table S9**.

CEGMA and Sequence Data Validation

CEGMA v2.4.010312 [10] as used to check for the existence of core eukaryotic genes (CEGs) in both the genome and transcriptome assemblies. Default parameters were used for the genome assembly, while --max_intron 0 was used for the transcriptome assembly. In order to assess the

validity of the final assembly, the CEGMA (Core Eukaryotic Genes Mapping Approach) [20] was used to establish our coverage of core eukaryotic genes (CEGs). Out of 248 CEGs, the ALLPATHS assembly included 218 completely assembled genes, with an additional 21 CEGs partially assembled, giving us an estimated gene completeness of 96% (239/248). We also had the genome assembly validated by the National Center for Biotechnology Information (NCBI), where it was checked for adaptors, primers, and low-complexity regions. The genome assembly has been approved and given the accession number JRLE00000000 and Bioproject PRJNA259363, and all the RNA-sequencing data has been deposited in the Sequence Read Archive (SRA, ID:264998).

MAKER annotation

Annotation was performed using MAKER v2.28 [11] following a two-pass workflow (https://github.com/sujaikumar/assemblage/blob/master/README-annotation.md). The workflow can be summarized as follows. First, CEGMA was run on the Metassembler-produced assembly. The CEGMA results were used for training a SNAP v2006-07-28 [12] hidden Markov model (HMM). Specifically, the cegma2zff program was run on the output cegma gff file. The fathom program was run with the genome.ann and genome.dna files produced by cegma2zff and a -categorize value of 1000. Fathom was run a second time with an -export value of 1000 and -plus inputs of uni.ann and uni.dna from the previous fathom step. The resulting export.ann and export.dna files were used as import to the forge program. The CEGMA/SNAP HMM was produced with the hmm-assembler.pl program. Next, a second HMM was produced using GeneMark-ES v2.3e [13,14] with default settings. In order to provide protein evidence for the MAKER annotation, we retrieved all protein sequences from the only other insofar published and publicly released hemipteran insect genome sequence (pea aphid *Acyrthosiphon pisum* assembly v2.1b https://www.aphidbase.com/aphidbase/content/download/3347/34150/file/aphidbase_2.1b_pep.fasta.bz2) [15]. The maker_opts.ctl file was edited to include the Metassembler genome assembly for the genome entry, the Trinity assembly for the est entry, the *Acyrthosiphon pisum* protein sequences for the protein entry, the CEGMA/SNAP HMM file for the snaphmm entry, the GeneMark-ES HMM file for the gmhmm entry, est2genome set to 1, protein2genome set to 1, keep_preds set to 1, and single_exon set to 1. The first iteration of MAKER was run with these configuration values. The MAKER program gff3_merge was used to merge together the resulting gff3 files from MAKER. This merged gff3 file was used as input to SNAP to build a second HMM using the SNAP HMM creation process as described previously. The genome.ann zff file created as part of the SNAP HMM creation process was used to generate a gff3 file using the zff2gff3 program included in the SNAP distribution. The Perl one-liner perl -plne ‘s/\t(\S+)$/\t\.\t$1/’ was used to add an extra column to the generated gff3 file for input to Augustus. An altered version of the autoAug.pl script from Augustus v2.7 [16] was used to generate an Augustus HMM which used a GMAP v2014-02-28 [17] alignment as a replacement for the BLAT [18] alignment used in the autoAug.pl pipeline. The parameters used for autoAug.pl were the genome assembly for the --genome argument, the transcript assembly for the --cdna argument, the gff3 file produced by SNAP for the --trainingset argument, --singleCPU, -v, and --useexisting. The GMAP alignment was generated by first running gmap_build on the genome assembly. The gmap command was then used to align the cdna.fa sequences (generated by autoAug.pl) to the indexed genome with the following parameters based on the BLAT alignment parameters from autoAug.pl: --min-identity=0.8 -B 5 --nthreads=10 --intronlength=100000 --format=psl. A second iteration of MAKER was run with the same parameters as described before with the following changes: the Augustus gene species model produced by autoAug.pl for the augustus_species entry, the second SNAP HMM was used for the

snaphmm parameter, est2genome set to 0, protein2genome set to 0, pred_stats set to 1, min_protein set to 30, alt_splice set to 1, split_hit set to 4000, single_length set to 250, and evaluate set to 1. The resulting gff3 files were merged using gff3_merge. Finally, the merged gff3 file was filtered by removing mRNAs along with their associated child features with an AED score <1 using Perl in-house developed scripts, grep, and fgrep.pl (https://github.com/sujaikumar/assemblage/blob/master/fgrep.pl). The resulting gff3 file was analyzed using a custom Python script making use of gffutils (http://pythonhosted.org/gffutils), numpy [19], and matplotlib [20] libraries to extract the relevant annotation information.

Gene model sequence extraction

Gene model sequences were extracted using the scaffold fasta file generated by Metassembler and the gff3 file generated by MAKER as input to the bedtools v2-2.19.1 [21] program getfasta.

Assembly contamination investigation

The Trinity transcriptome assembly was aligned to human reference genome version hg19 using the STAR v2.3.1z aligner [22]. In order to accommodate the longer lengths of the transcript sequences (compared to RNA sequencing read lengths), STAR was compiled with the STARlong option. STAR was run using the following parameters: --outFilterMismatchNmax 100 --seedSearchLmax 30 --seedSearchStartLmax 30 --seedPerReadNmax 100000 --seedPerWindowNmax 100 --alignTranscriptsPerReadNmax 100000 --alignTranscriptsPerWindowNmax 10000. Mapped reads were filtered from the resulting SAM file using samtools with the -F 4 option. The alignment length, as well as the total edit distance as reported by the "NM" tag of the remaining alignments were extracted from the SAM file using awk. This information was used to calculate the percentage identity of the aligned sequence. The meta-assembly of the genome was aligned to a local copy of the RefSeq [23] human_genomic database (downloaded on May 7, 2014) using BLASTN 2.2.28+ [24] with the following parameters: -outfmt 6 -num_threads 20 -max_target_seqs 10 -evalue 0.001. The awk program was used to filter the BLASTN results by alignment length and percent identity. After submitting the Metassembler-based genome assembly to NCBI, a contamination screen identified regions of the scaffolds that were flagged as contaminated due to the presence of sequences of known primers or other organisms. These sequences were removed and the containing scaffolds were split. The identifier code of the scaffold was retained and a segment identifier was created based on the number of sequences resulting from the contamination removal. New identifier codes were created by appending segment identifiers to the original scaffold identifier code separated by a period (.).

Gene expression analysis

Single-end 50 bp Illumina reads from each developmental stage (1st, 2nd, 3rd, 4th, and 5th instar nymphs) and adult (male and female) were aligned to the meta-assembly of the genome. First, the genome was indexed using the genomeGenerate mode of the STAR aligner using the following parameters: --runThreadN 20 --sjdbGTFfile bedbug.v1.gff --sjdbGTFtagExonParentTranscript Parent --sjdbOverhang 99, where the gff file was generated by the two-pass MAKER annotation described previously. In order to avoid having gene model names truncated when mapping RNA-seq reads to the genome, the names were shortened to provide a unique, short name for each gene. Each set of RNA-seq reads from the developmental stages and adult sex groups were aligned to the indexed genome sequence using STAR with the following parameters: --readFilesCommand zcat --runThreadN 20 --outReadsUnmapped Fastx. STAR produced alignments in SAM format for each set of RNA-seq data. Each of these SAM files was converted to a BAM file using samtools using

the view command with parameters -Sb. Each BAM file was then sorted using the samtools sort command. In order to perform pairwise differential expression analysis for each RNA-seq dataset, the MAKER-generated gff file and BAM files were uploaded to the Rätsch Lab Galaxy [25] server (https://galaxy.cbio.mskcc.org). Pairwise differential expression analysis was performed using the DESeq2 v1.0.19 [26] Galaxy wrapper which is integrated into the Rätsch Lab's Online Quantitative Transcriptome Analysis (Oqtans) tool suite (http://oqtans.org). Each RNA-seq group (instars and sex) was submitted as a replicate group with each replicate being submitted individually. For the Select fitting to the mean intensity parameter, the mean option was chosen. The job was submitted resulting in a tab-delimited file of DEG models. DEGs were filtered using an absolute fold-change cutoff of ≥1.5 and a Benjamini-Hochberg adjusted *P*-value of ≤0.05 to produce a set of DEGs for each pairwise comparison.

Functional annotation

We performed functional annotation of bedbug sequences based on the gene ontology (GO) vocabulary using the Blast2GO v2.5.0 pipeline (https://www.blast2go.com) with the following parameters: java -Xmx50G -cp *:ext/*: es.blast2go.prog.B2GAnnotPipe -in bedbug.allBBgeneMatches.txt -out bedbug_out_50G.annot -prop b2gPipe.properties.local –annot, where b2gPipe.properties.local points to a local Blast2GO database. We also used InterProScan v5.5-48.0 [27] with the following parameters: -dp -f TSV,XML,GFF3 -goterms -iprlookup -i Cimex_lectularius.

Human contamination of RNA-seq data

Unaligned reads retained when producing previously described RNA-seq alignments to the Metassembler genome assembly were aligned to human genome hg19 using STAR. The samtools view command was used to count aligned reads with the -S -c -F 4 options.

Active gene discovery

Sorted bam files for each developmental stage and sex as described previously were used as input to the rpkmforgenes.py program [28]. Each replicate bam file was processed separately. The resulting RPKM values were filtered at three different RPKM thresholds: 0.1, 1, and 10. A gene model is only considered active in the case that RPKM values for all three replicates surpassed the threshold. The counts for genes considered active were plotted using Python's matplotlib.

Analysis of genes related to blood-feeding activity

Several suites amino-acid sequences from anticoagulants and other bioactive proteins involved blood feeding known from other sanquivorous taxa were prepared as target databases for blastp searches using unannotated predicted gene products from the combined Qmolecula/allpaths hybrid assembly. Those targeted were anti-thrombins, factor Xa inhibitors, platelet aggregation and activation inhibitors, hyaluronidases and plasminogen activators. In addition, the full set of predicted gene products was compared both to ToxProt, a compilation of all toxin proteins produced by venomous animals, as well as a third query database comprising all salivary protein sequences already annotated for Cimicomorpha at NCBI. The latter consists primarily of those sequences available for the saliome of Tratima infestans. High-scoring matches (e-value <-60) then were sorted and evaluated for relevance to salivary and blood-feeding related functionality. Premised on the notion that to be biologically active in the context of sangivoury activity, and that they would be expected to be targeted to the extracellular environment, amino acid sequences were subject to prediction of N-terminal signal peptide regions (D-cutoff = 0.50) leveraging artificial neural

network systems through SignalP 4.1 at http://www.cbs.dtu.dk/services/SignalP/. Predicted gene products were then compiled and compared with BLASTP against the full suite of available annotated sequences (NR in GenBank) to determine whether another non-target functionality was a better match; if a better e-value was found these were removed.

We mined the set of bedbug protein sequences via BLASTP by using as queries a multitude of proteins from other species known to confer partial or full resistance to insecticidal compounds, when (1) containing one or more amino acid replacements, (2) their genes are duplicated, or (3) their genes are associated with transposable elements. The bedbug hits were queried themselves against the UniProt protein knowledgebase (http://www.uniprot.org) using BLASTP, and the results were manually inspected for similarity to candidates of known function.

Bacterial genetic traces

We downloaded all of the complete bacterial genomes that were listed in Ensembl release 24 (ftp://ftp.ensemblgenomes.org/pub/release-24/bacteria/fasta). In total, this sample included 20,030 bacterial strains. We ran reciprocal TBLASTX searches between the bacterial genomes and both the *C. lectularius* gene set and the full genome sequence using a cutoff E-value of <1e-5 and required a 30 bp overlap match. For the SNP calling, we ran MUMmer [29] to compare the gene calls from the bedbug genome against the reference *C. lectularius Wolbachia* endosymbiont (*w*Cle) genome [30].

Protein modeling

Protein structural modeling was carried out with SWISS-MODEL (http://swissmodel.expasy.org) producing a high quality structure with a model-template C-α root mean square deviation of 2.3 Å. The models were further refined with Molecular Dynamics (MD) simulations with the Amber14 molecular dynamics suite [31]. The proteins and ATP molecules were placed in a water box, and after initial minimization and equilibration for 1 ns, the production run with the canonical (NVT) ensemble and Langevin thermostat heat exchange totaling 100 ns was conducted on a high-performance Linux cluster with NVIDIA Tesla GPU nodes. MD trajectory files were collected and an average structure over all 100-ns time frames was calculated for each model with the VMD program [32] and followed by a brief minimization. Post MD simulation analysis and visual representations were conducted in MOE program [33].

All available 39 X-ray crystal structures of DDL proteins were downloaded from the Protein Data Bank (http://www.rcsb.org). After aligning protein sequences we searched for the residues that were located in the same positions as in the reported network, and indeed found substantial supporting evidence for such network occurrence. Among these 39 structures, 24 of them have lysine in the position similar to K168 of *Wolbachia*. Aspartic acid in position 96 is conserved among 38 available crystal structures. There are some variations in position 98, where we also observed a mutation A98T. Aspartic acid is the most common amino acid in this position (occurred 15 times), followed by leucine (also 15 times). There is no available crystal structure of DDL with threonine in position 98 (**Table S8**). Interestingly, three member networks similar to the D96-T98-K168 hydrogen-bonding network observed after MD simulations in the K168 mutant form of *Wolbachia* were present in all D96-D98-K168 and D96-L98-K168 X-ray crystal structures. However, if K168 is replaced with E, as happens in 10 crystal structures, then such network is not observed. It is especially evident for sequences where position 98 is occupied by amino acids with aliphatic side chains, e.g. leucine. We found it very intriguing that such hydrogen bond network occurred only in the mutant protein despite the fact that our template structures, 1IOV and 4C5B,

lack this network. As we mentioned in the manuscript, the replacement of alanine with the larger threonine sidechain which can serve as a hydrogen bond donor, may help the formation of this three member network T98-D96-K168 and facilitate the shift of T98 toward K168 in the mutant protein that resulted in 95-98 strand shift and create more space for ATP binding in the mutant DDL vs wild type A98 DDL.

Based on the computational model we concluded that among eight observed mutations, A58D, I60V, T84R, I93V, A98T, L104F, G108D, I109V, none was directly involved into the binding of ATP. However it is worth noting that, in the wild-type protein, the residues in positions 58, 60 and 84 are in close proximity and form a hydrogen-bonding network that stabilizes loops formation in this region. It was expected that a change from a small neutral residue to a larger charged residue (e.g. A58D, T84R) might cause reorganization of the loops. The comparison of the wild type and mutant DDL models suggests that a replacement to oppositely charged amino acids may lead to stronger interactions within this network. In addition to hydrogen bonds, strong ionic interactions occur between D58 and R84 in the mutant protein. This in turn leads to partial changes in adjacent flexible regions as seen in **Supplementary Figure 10** and may cause some alteration in ligase activity.

Evolutionary relationships

We established 1:1 orthology relationships with another 19 arthropod fully sequenced genomes using a combination of sequence similarity and clustering procedures as well as phylogenetic criteria as implemented in the OrthologID pipeline[34,35]. We then analyzed all orthologs in a phylogenetic framework in two ways. We constructed a gene content framework for bedbug in the context of 20 other fully sequenced arthropod genomes by combining orthologous loci according to their presence (character coded as 1) or absence (character coded as 0). We analyzed this presence-absence matrix using our Venninator program[36,37]. The gene content phylogenetic matrix was analyzed using equally weighted and Dollo parsimony in PAUP* 4.0b10 (http://paup.csit.fsu.edu), as well as with maximum likelihood (ML) phylogenetic inference using the BINGAMMA model in the POSIX-threads build of RAxML v8[38]. The protein supermatrix was analyzed using maximum likelihood in RAxML with a general time-reversible (GTR) substitution matrix estimated from our arthropod proteomic sequences. We contrasted the fit of our data-derived GTR substitution model to the commonly used WAG model [39]. The empirical residue frequencies were used and the among-site rate heterogeneity was modeled using the Γ distribution and four discrete rate categories [40]. Node robustness was assessed via bootstrap resampling [41].

The ddl sequences from all *Wolbachia* genomes from insects were downloaded from NCBI GenBank and aligned by respecting the protein-coding frame using TranslatorX [42]. The final alignment of 14 sequences was trimmed to match the length of the bedbug *ddl* sequence (951 bp, 317 aa). The *Brugia malayi* (nematode) *Wolbachia* was set as outgroup. Phylogenetic tree inference was carried out using both Maximum Parsimony (MP) and ML in PAUP and RAxML. ML inference was run using the general time-reversible (GTR) nucleotide substitution model and the Γ distribution and four discrete rate categories. The ML and MP trees were identical with very similar bootstrap node support values. We analyzed codon by codon selection by contrasting the rates of fixation of nonsynonymous (dN) vs. synonymous (dS) substitutions in Datamonkey (http://www.datamonkey.org) using various models: MEME (mixed effects model evolution) which can identify codons undergoing episodic or pervasive selection, FEL (fixed effects likelihood) that

directly estimates dN and dS at each codon and SLAC (single ancestor likelihood counting), which is the most conservative method contrasting dN and dS rates, and FUBAR (Fast Unconstrained Bayesian AppRoximation), a robust method that can detect codons experiencing positive and purifying selection. Furthermore, we examined the potential for diversifying selection to have acted on internal branches of the *ddl* genealogy using the branch-site model implemented in BSREL (branch-site random effects likelihood). In all cases the ML gene tree was used as guide tree.

Signal peptide detection

We used the program SignalP v4.0[43] [ref] to identify evidence of signal peptides in the proteins. Strong evidence of a signal peptide sequence was considered a D-score exceeding the dynamically determined threshold value (typically 0.45 or 0.5).

Metagenomic sampling

The metagenomic samples were obtained from the PathoMap project (http://www.pathomap.org) [44] and the reads from 1,447 sampled New York City subway locations were aligned against the *C. lectularius* genome sequence using BWA[45]. Variants were called using freebayes [46] and manipulated using PLINK[47] in order to produce a subset with calls for 90% of the locations. We then constructed a phylogenetic tree using MP and a heuristic search with TBR (tree bisection-reconnection) branch swapping and 100 random additions as starting points in PAUP. A retention index (RI) was calculated for the given the phylogeny. One-tailed randomization tests for each variable tested whether or not the actual RI was significantly greater than the RIs of randomized data. Randomized RI data were calculated by randomizing the characters ascribed to terminals for each variable and then determining their RI given the SNP phylogeny (9,999 replicates). Randomization tests were conducted using R with the packages APE [48] and phangorn[49].

We mapped the resulting phylogenetic trees on a two-dimensional geographical map using the GPS coordinates of the sampled subway locations. The tree files and latitude-longitude coordinates were converted to .kml format files with the GeoPhylo Engine[50], and were examined in Google Earth (https://www.google.com/earth).

Anticoagulant Gene Analysis

We gathered a collection of anticoagulants from a wide range of species and using BLAST ad compared them to the bedbug proteome. High-scoring matches (D-score <0.50) for predicted gene products with complete signal peptide secretory sequences were found for the serine protease inhibitor infestin, the antihemostatic (anti-platelet aggregation factor) apyrase, and the vasodilator or anti-histamine lipocalin, all three of which are the result of adaptations to blood feeding. More specifically, infestin is a Kazal-type thrombin inhibitor (binding in a slow, tight-binding, competitive process) that is utilized as a structural scaffold template for exogenous anticoagulants [51]. Infestin is found in the kissing bug Triatoma infestans. Apyrase, which may promote the formation of hematomas, is a salivary enzyme (ATP-diphosphohydrolase) that hydrolyzes ATP and ADP to AMP and orthophosphate, thus preventing the effect of ADP on hemostasis (ADP is an important stimuli for platelet aggregation in vertebrates) [52]. The thrombin and intrinsic tenase complex (ITC) inhibitor lipocalin has a characteristic eight-stranded anti-parallel β-barrel structure that the kissing bug Triatoma pallidipennisuses as a scaffold for anticoagulants [53]. Lipocalin is also found in the kissing bug Rhodnius prolixus. We also found for a variety of characterized proteins with less obvious associations to a blood feeding lifestyle. Venom metalloproteases are most intensively studied in the context of crotaline and viperine snake envenomations wherein their

hemorrhagic activity relates to endothelial pathology, fibrinogenolysis and their ability to act as disintegrins that inhibit platelet aggregation [54]. Zinc-binding metalloproteases are present in the saliomic profiles of a wide range of arthropod sanguivores, including ticks [55], hookworms [56] and cimicomorphs related to bedbugs; e.g., the reduviids [57]. Serine protease inhibitors are more commonly associated with a blood feeding habit than are serine proteases [58]. Nonetheless, a variety of these proteases and other trypsin-like plasminogen activators have been characterized from the salivary transcriptomic profiles of the relatively closely related Triatoma matogrossensis and Triatoma infestans [59]. These references were all used for the comparison to the bedbug proteome and genome.

The raw sequences used to generated the tree were:
gi|115392217|gb|ABI96910.1| brasiliensin precursor [Triatoma brasiliensis]
gi|118137638|pdb|2ERW|A Chain A, Crystal Structure Of Infestin 4, A Factor Xiia Inhibitor
gi|14211145|gb|AAK57342.1| thrombin inhibitor infestin precursor, partial [Triatoma infestans]
gi|14211145|gb|AAK57342.1| thrombin inhibitor infestin4 precursor, partial [Triatoma infestans]
gi|167871104|gb|EDS34487.1| serine protease inhibitor dipetalogastin [Culex quinquefasciatus]
gi|170049257|ref|XP_001855099.1| serine protease inhibitor dipetalogastin [Culex quinquefasciatus]
gi|193683435|ref|XP_001945453.1| PREDICTED: serine protease inhibitor dipetalogastin [Acyrthosiphon pisum]
gi|307180124|gb|EFN68168.1| Serine protease inhibitor dipetalogastin [Camponotus floridanus]
gi|332019031|gb|EGI59565.1| Serine protease inhibitor dipetalogastin [Acromyrmex echinatior]
gi|357614659|gb|EHJ69197.1| putative serine protease inhibitor dipetalogastin precursor [Danaus plexippus]
gi|4033530|emb|CAA10384.1| dipetalogastin [Dipetalogaster maximus]
gi|405975560|gb|EKC40118.1| Serine protease inhibitor dipetalogastin [Crassostrea gigas]
gi|485220029|gb|JAA76439.1| putative 3-kazal and poly his protein similar to brasiliensin precursor [Rhodnius prolixus]
gi|485221363|gb|JAA77097.1| putative multi kazal and poly-his protein similar to brasiliensin, partial [Rhodnius prolixus]
gi|485221649|gb|JAA77239.1| putative similr to brasiliensin precursor, partial [Rhodnius prolixus]
gi|512898569|ref|XP_004924430.1| PREDICTED: serine protease inhibitor dipetalogastin [Bombyx mori]
gi|550239047|gb|JAB62011.1| Serine protease inhibitor dipetalogastin, partial [Anoplophora glabripennis]
gi|577744249|gb|JAC03763.1| Serine protease inhibitor dipetalogastin [Ceratitis capitata]
gi|604774863|gb|JAC09882.1| putative cpij010521 serine protease inhibitor dipetalogastin [Aedes albopictus]
gi|642929560|ref|XP_975339.2| PREDICTED: serine protease inhibitor dipetalogastin [Tribolium castaneum]
gi|645016105|ref|XP_008211344.1| PREDICTED: serine protease inhibitor dipetalogastin isoform X4 [Nasonia vitripennis]
gi|749781027|ref|XP_011144857.1| PREDICTED: serine protease inhibitor dipetalogastin [Harpegnathos saltator]
gi|751453682|ref|XP_011181276.1| PREDICTED: serine protease inhibitor dipetalogastin isoform X2 [Bactrocera cucurbitae]
gi|755657405|gb|JAG73077.1| Serine protease inhibitor dipetalogastin, partial [Fopius arisanus]

gi|769834463|ref|XP_011647333.1| PREDICTED: serine protease inhibitor dipetalogastin [Pogonomyrmex barbatus]
gi|780042099|ref|XP_011668235.1| PREDICTED: serine protease inhibitor dipetalogastin isoform X2 [Strongylocentrotus purpuratus]

Bayesian phylogenetic inference was also performed (lset rates=gamma; prset aamodelpr = mixed; mcmc ngen=1,000,000; sumt burnin=200,000). The Bayesian tree was in broad agreement with the MP tree.

Accession Codes

The genome assembly has been approved and given the accession number JRLE00000000 and BioProject PRJNA259363. All genome sequencing data has been deposited in the Sequence Read Archive (SRA) with accession number SRS749263. RNA-seq data is available as FASTQ files and were quality-checked and deposited in the SRA with accession SRR1790655.

Supplementary References

1. Kvist S, Brugler MR, Goh TG, Giribet G, Siddall ME (2014) Pyrosequencing the salivary transcriptome of Haemadipsa interrupta (Annelida: Clitellata: Haemadipsidae): anticoagulant diversity and insight into the evolution of anticoagulation capabilities in leeches. Invertebrate Biology 133: 74-98.
2. Simpson JT, Wong K, Jackman SD, Schein JE, Jones SJ, et al. (2009) ABySS: a parallel assembler for short read sequence data. Genome research 19: 1117-1123.
3. Koren S, Schatz MC, Walenz BP, Martin J, Howard JT, et al. (2012) Hybrid error correction and de novo assembly of single-molecule sequencing reads. Nature biotechnology 30: 693-700.
4. Schatz M (2001) Metassembler.
5. Zhang M, Zhang Y, Scheuring CF, Wu C-C, Dong JJ, et al. (2012) Preparation of megabase-sized DNA from a variety of organisms using the nuclei method for advanced genomics research. nature protocols 7: 467-478.
6. Cao H, Hastie A, Cao D, Lam E, Sun Y, et al. (2014) Rapid detection of structural variation in a human genome using nanochannel-based genome mapping technology. GigaScience 3: 34.
7. Haas BJ, Papanicolaou A, Yassour M, Grabherr M, Blood PD, et al. (2013) De novo transcript sequence reconstruction from RNA-seq using the Trinity platform for reference generation and analysis. Nature protocols 8: 1494-1512.
8. Lam ET, Hastie A, Lin C, Ehrlich D, Das SK, et al. (2012) Genome mapping on nanochannel arrays for structural variation analysis and sequence assembly. Nature biotechnology 30: 771-776.
9. Cox MP, Peterson DA, Biggs PJ (2010) SolexaQA: At-a-glance quality assessment of Illumina second-generation sequencing data. BMC bioinformatics 11: 485.
10. Parra G, Bradnam K, Korf I (2007) CEGMA: a pipeline to accurately annotate core genes in eukaryotic genomes. Bioinformatics 23: 1061-1067.
11. Holt C, Yandell M (2011) MAKER2: an annotation pipeline and genome-database management tool for second-generation genome projects. BMC bioinformatics 12: 491.
12. Korf I (2004) Gene finding in novel genomes. BMC bioinformatics 5: 59.
13. Lomsadze A, Ter-Hovhannisyan V, Chernoff YO, Borodovsky M (2005) Gene identification in novel eukaryotic genomes by self-training algorithm. Nucleic Acids Research 33: 6494-6506.
14. Ter-Hovhannisyan V, Lomsadze A, Chernoff YO, Borodovsky M (2008) Gene prediction in novel fungal genomes using an ab initio algorithm with unsupervised training. Genome research 18: 1979-1990.
15. Consortium IAG (2010) Genome sequence of the pea aphid Acyrthosiphon pisum. PLoS biology 8: e1000313.
16. Stanke M, Waack S (2003) Gene prediction with a hidden Markov model and a new intron submodel. Bioinformatics 19: ii215-ii225.
17. Wu TD, Watanabe CK (2005) GMAP: a genomic mapping and alignment program for mRNA and EST sequences. Bioinformatics 21: 1859-1875.
18. Kent WJ (2002) BLAT—the BLAST-like alignment tool. Genome research 12: 656-664.
19. Oliphant TE (2007) Python for scientific computing. Computing in Science & Engineering 9: 10-20.
20. Hunter JD (2007) Matplotlib: A 2D Graphics Environment. Computing in Science and Engg 9: 90-95.
21. Quinlan AR, Hall IM (2010) BEDTools: a flexible suite of utilities for comparing genomic features. Bioinformatics 26: 841-842.

22. Dobin A, Davis CA, Schlesinger F, Drenkow J, Zaleski C, et al. (2013) STAR: ultrafast universal RNA-seq aligner. Bioinformatics 29: 15-21.
23. Pruitt KD, Brown GR, Hiatt SM, Thibaud-Nissen F, Astashyn A, et al. (2014) RefSeq: an update on mammalian reference sequences. Nucleic acids research 42: D756-D763.
24. Altschul SF, Gish W, Miller W, Myers EW, Lipman DJ (1990) Basic local alignment search tool. Journal of molecular biology 215: 403-410.
25. Blankenberg D, Kuster GV, Coraor N, Ananda G, Lazarus R, et al. (2010) Galaxy: a web-based genome analysis tool for experimentalists. Current protocols in molecular biology: 19.10. 11-19.10. 21.
26. Love MI, Huber W, Anders S (2014) Moderated estimation of fold change and dispersion for RNA-Seq data with DESeq2. bioRxiv.
27. Jones P, Binns D, Chang H-Y, Fraser M, Li W, et al. (2014) InterProScan 5: genome-scale protein function classification. Bioinformatics 30: 1236-1240.
28. Ramsköld D, Wang ET, Burge CB, Sandberg R (2009) An abundance of ubiquitously expressed genes revealed by tissue transcriptome sequence data. PLoS computational biology 5: e1000598.
29. Kurtz S, Phillippy A, Delcher AL, Smoot M, Shumway M, et al. (2004) Versatile and open software for comparing large genomes. Genome biology 5: R12.
30. Nikoh N, Hosokawa T, Moriyama M, Oshima K, Hattori M, et al. (2014) Evolutionary origin of insect–Wolbachia nutritional mutualism. Proceedings of the National Academy of Sciences 111: 10257-10262.
31. D.A. Case JTB, R.M. Betz, D.S. Cerutti, T.E. Cheatham, III, T.A. Darden, R.E. Duke, T.J. Giese, H. Gohlke, A.W. Goetz, N. Homeyer, S. Izadi, P. Janowski, J. Kaus, A. Kovalenko, T.S. Lee, S. LeGrand, P. Li, T. Luchko, R. Luo, B. Madej, K.M. Merz, G. Monard, P. Needham, H. Nguyen, H.T. Nguyen, I. Omelyan, A. Onufriev, D.R. Roe, A. Roitberg, R. Salomon-Ferrer, C.L. Simmerling, W. Smith, J. Swails, R.C. Walker, J. Wang, R.M. Wolf, X. Wu, D.M. York and P.A. Kollman (2015) AmberTools15. University of California, San Francisco: University of California, San Francisco.
32. Humphrey W, Dalke A, Schulten K (1996) VMD: visual molecular dynamics. Journal of molecular graphics 14: 33-38.
33. (2013) Molecular Operating Environment (MOE). 1010 Sherbooke St. West, Suite #910, Montreal, QC, Canada, H3A 2R7, 2015: Chemical Computing Group Inc., .
34. Egan M, Lee EK, Chiu JC, Coruzzi G, DeSalle R (2009) Gene orthology assessment with OrthologID. Bioinformatics for DNA Sequence Analysis: Springer. pp. 23-38.
35. Chiu JC, Lee EK, Egan MG, Sarkar IN, Coruzzi GM, et al. (2006) OrthologID: automation of genome-scale ortholog identification within a parsimony framework. Bioinformatics 22: 699-707.
36. Lienau EK, DeSalle R, Rosenfeld JA, Planet PJ (2006) Reciprocal illumination in the gene content tree of life. Systematic biology 55: 441-453.
37. Rosenfeld JA, DeSalle R, Lee EK, O'Grady P (2008) Using whole genome presence/absence data to untangle function in 12 Drosophila genomes. Fly 2: 291-299.
38. Stamatakis A (2014) RAxML version 8: a tool for phylogenetic analysis and post-analysis of large phylogenies. Bioinformatics 30: 1312-1313.
39. Whelan S, Goldman N (2001) A general empirical model of protein evolution derived from multiple protein families using a maximum-likelihood approach. Mol Biol Evol 18: 691-699.
40. Yang Z (1994) Maximum likelihood phylogenetic estimation from DNA sequences with variable rates over sites: approximate methods. J Mol Evol 39: 306-314.

41. Felsenstein J (1985) Confidence limits on phylogenies: an approach using the bootstrap. Evolution: 783-791.
42. Abascal F, Zardoya R, Telford MJ (2010) TranslatorX: multiple alignment of nucleotide sequences guided by amino acid translations. Nucleic acids research: gkq291.
43. Petersen TN, Brunak S, von Heijne G, Nielsen H (2011) SignalP 4.0: discriminating signal peptides from transmembrane regions. Nature methods 8: 785-786.
44. Afshinnekoo E, Meydan C, Chowdhury S, Jaroudi D, Boyer C, et al. (2015) Geospatial Resolution of Human and Bacterial Diversity with City-Scale Metagenomics. Cell Systems.
45. Otti O, Naylor RA, Siva-Jothy MT, Reinhardt K (2009) Bacteriolytic activity in the ejaculate of an insect. The American Naturalist 174: 292-295.
46. Garrison E, Marth G (2012) Haplotype-based variant detection from short-read sequencing. arXiv preprint arXiv:12073907.
47. Purcell S, Neale B, Todd-Brown K, Thomas L, Ferreira MA, et al. (2007) PLINK: a tool set for whole-genome association and population-based linkage analyses. The American Journal of Human Genetics 81: 559-575.
48. Paradis E, Claude J, Strimmer K (2004) APE: analyses of phylogenetics and evolution in R language. Bioinformatics 20: 289-290.
49. Schliep KP (2011) phangorn: Phylogenetic analysis in R. Bioinformatics 27: 592-593.
50. Hill AW, Guralnick RP (2010) GeoPhylo: an online tool for developing visualizations of phylogenetic trees in geographic space. Ecography 33: 633-636.
51. Koh, C.Y. and R.M. Kini, *Molecular diversity of anticoagulants from haematophagous animals.* Thromb Haemost, 2009. **102**(3): p. 437-453.
52. Ribeiro, J., *Role of saliva in blood-feeding by arthropods.* Annual review of entomology, 1987. **32**(1): p. 463-478.
53. Andersen, J.F., et al., *The role of salivary lipocalins in blood feeding by Rhodnius prolixus.* Archives of insect biochemistry and physiology, 2005. **58**(2): p. 97-105.
54. Gutiérrez, J.M. and A. Rucavado, *Snake venom metalloproteinases:Their role in the pathogenesis of local tissue damage.* Biochimie, 2000. **82**(9–10): p. 841-850.
55. Francischetti, I., T.N. Mather, and J. Ribeiro, *Cloning of a salivary gland metalloprotease and characterization of gelatinase and fibrin (ogen) lytic activities in the saliva of the Lyme disease tick vector< i> Ixodes scapularis</i>.* Biochemical and biophysical research communications, 2003. **305**(4): p. 869-875.
56. Feng, J., et al., *Molecular cloning and characterization of< i> Ac</i>-MTP-2, an astacin-like metalloprotease released by adult< i> Ancylostoma caninum</i>.* Molecular and biochemical parasitology, 2007. **152**(2): p. 132-138.
57. Assumpção, T.C., et al., *An insight into the sialotranscriptome of Triatoma matogrossensis, a kissing bug associated with fogo selvagem in South America.* The American journal of tropical medicine and hygiene, 2012. **86**(6): p. 1005-1014.
58. Fry, B.G., et al., *The toxicogenomic multiverse: convergent recruitment of proteins into animal venoms.* Annual review of genomics and human genetics, 2009. **10**: p. 483-511.
59. Amino, R., A.S. Tanaka, and S. Schenkman, *Triapsin, an unusual activatable serine protease from the saliva of the hematophagous vector of Chagas' disease< i> Triatoma infestans</i>(Hemiptera: Reduviidae).* Insect biochemistry and molecular biology, 2001. **31**(4): p. 465-472.
